# Supplementary material for: Health workforce for oral health inequity: Opportunity for action
Source: PLoS One. 2024 Jun 13;19(6):e0292549. doi: 10.1371/journal.pone.0292549 (PMC11175420; doi:10.1371/journal.pone.0292549)
Supplement: S1 Text — (DOCX) [file pone.0292549.s001.docx]

**Supporting Information 1: Definition of oral health workforce categories**

| Dentist | Dentists diagnose, treat and prevent diseases, injuries and abnormalities of the teeth, mouth, jaws and associated tissues by applying the principles and procedures of modern dentistry. They use a broad range of specialised diagnostic, surgical and other techniques to promote and restore oral health. |
| --- | --- |
| Dental Assistants and Therapists | Dental assistants and therapists provide basic dental care services for the prevention and treatment of diseases and disorders of the teeth and mouth, according to care plans and procedures established by a dentist or other oral health professional. Under this dental assistants and therapists, dental hygienist and dental nurse will be covered. |
| Dental Prosthetic/Technician | Dental prosthetic technicians design, fit, service and repair dental devices and appliances following prescriptions or instructions established by a health professional. They may service a wide range of support instruments to correct dental problems, such as dentures, and dental crowns and bridges. This will include clinical and non-clinical technicians. |

Source: International Standard Classification of Occupations (ISCO-08), 2012. S*tructure, group definitions and correspondence tables*, viewed 7 June 2021, [https://www.ilo.org/wcmsp5/groups/public/---dgreports/---dcomm/---publ/documents/publication/wcms_172572.pdf].

Glossary of common terms related to oral health workforce

| Mid-level oral health workforce providers | Mid-level oral health workers are those who have received shorter training than dentists (between 2–4 years) but will perform some of the same tasks as dentists. Therefore, a mid-level oral health worker is not a clinical dentist but is able to provide clinical care (may diagnose, manage and treat disease and impairments) or engage in preventive care and health promotion. The examples are dental assistants, dental nurses, dental prosthetists, dental therapists and dental hygienists. |
| --- | --- |

Sources: Lassi ZS, Cometto G, Huicho L, Bhutta ZA. Quality of care provided by mid-level health workers: systematic review and meta-analysis. *Bull World Health Organ*. 2013;91(11):824-33I.

World Health Organization, 2018. Mid-level health workers: a review of the evidence. <https://apps.who.int/iris/handle/10665/259878>.

World Health Organization. Political declaration of the third high-level meeting of the General Assembly on the prevention and control of noncommunicable diseases. World Health Organization; 2022 11/01/2022. Contract No.: EB150/7.

| Task shifting | Task shifting is defined as delegating tasks to existing or new health workers with either less training or narrowly tailored training. It involves the rational redistribution of tasks among health workforce teams. Specific tasks are moved, where appropriate, from highly qualified cadres to cadres with shorter training and fewer qualifications in order to make more efficient use of the available human resources for health. |
| --- | --- |

Sources: World Health Organization, 2008. Task shifting: Global recommendations and guidelines. *Geneva: World Health Organization.* Sep 4;1.

World Health Organization, 2018. Mid-level health workers: a review of the evidence. <https://apps.who.int/iris/handle/10665/259878>.

| Skill mix | Skill mix is a broad term that has been defined as the proportion of staff qualifications, levels of competence, abilities, knowledge and experience that are necessary to achieve an agreed standard of care for a given level of demand. It represents the mix of different types of staff in a team/healthcare setting. |
| --- | --- |

Sources: Cahill, J., 1995. Making the most of an essential resource. Using skill mix for the benefit of staff and patients*. Professional nurse (London, England*), 10(6), pp.355-358.

Buchan, J. and Dal Poz, M.R., 2002. Skill mix in the health care workforce: reviewing the evidence. *Bull World Health Organ*, 80(7), pp.575-580.
