# Supplementary material for: Health workforce for oral health inequity: Opportunity for action
Source: PLoS One. 2024 Jun 13;19(6):e0292549. doi: 10.1371/journal.pone.0292549 (PMC11175420; doi:10.1371/journal.pone.0292549)
Supplement: S2 Text — (DOCX) [file pone.0292549.s002.docx]

**Supporting Information 2: The WHO Global Oral Health Workforce Survey Questionnaire**

Start of Block: Introduction

The WHO Global Oral Health Workforce Survey

Purpose: The purpose of this survey is to gauge your country’s workforce information responding to oral health.  It will provide the global situation of oral health workforce to describe current workforce situation and provide clear picture how to leverage existing system (health workforce) to maximize population benefit, health and well-being at country level in the current global context, namely, Universal Health Coverage (UHC) and the 13th General Programme of Work towards the Sustainable Development Goals (SDGs).

- The information collected through this survey will be presented as part of the "Global Oral Health Report" which is planned to be launched in May 2020.
- The results of this survey will also be contributed to the WHO National Health Workforce Accounts.
- Use of standardized questions allows comparisons of country capacities and responses.
- We have divided this survey into four modules:

1. General information
2. Oral health workforce: capacity, capability and governance
3. Education and training of the oral health workforce
4. Better use of the oral health workforce

Process:

- A focal point or survey coordinate will need to be identified to coordinate and ensure survey completion. However, in order to provide a complete response, a group of respondents with expertise in the topics covered in the modules will be needed. Please use the table provided to indicate names and titles of all of those who have completed the survey and which sections they have completed. Please also add any additional information on other sources you may have consulted in developing your response.
- Please note that while there is space to indicated "Don't Know" for most questions, there should be very few of these. If someone is filling in numerous "Don't Knows", another person who is more aware of this information should be found to complete this section.
- The responses are automatically saved and you can return back to the responses within 3 months if you access it through the same URL.
- As far as possible, in order to validate responses, documentation will be requested for affirmative responses throughout the questionnaire. Please make every effort to provide electronic copies of the requested documentation by email (varenneb@who.int and makinoy@who.int). If you are unable to provide electronic copies through the email then please contact varenneb@who.int and makinoy@who.int for an alternative means to submit documentation. Please note that questionnaire survey terminology is aligned with the International Standard Classification of Occupations (ISCO-08) and the WHO National Health Workforce Accounts.

*[ISCO-08*: http://www.ilo.org/wcmsp5/groups/public/@dgreports/@dcomm/@publ/documents/publication/wcms_172572.pdf
https://www.ilo.org/public/english/bureau/stat/isco08/index.htm]

| Page Break |  |
| --- | --- |

**Glossary**

| Accreditation | Accreditation is a process by which an officially approved body, on the basis of assessment of learning outcomes and /or competences according to different purposes and methods, awards qualifications (certificates, diplomas or titles), or grants equivalences, credit units or exemptions, or issues documents such as portfolios of competences. In some cases, the term accreditation applies to the evaluation of the quality of an institution or a programme as a whole. |
| --- | --- |
| Active health worker | Active health workers are those who provide services for patients (practising health professionals). In case of data not available for practising health workers, data closest to practising (professionally active health workers, health workers with active license) can be used. |
| Community Health Workers | Community health workers provide health education, referral and follow-up, case management, basic preventive health care and home visiting services to specific communities. They provide support and assistance to individuals and families in navigating the health and social services system. |
| Continuing professional development | Training that is beyond clinical update and includes wide-ranging competencies like research and scientific writing; multidisciplinary context of patient care; professionalism and ethical practice;  communication, leadership, management and behavioural skills; team building; information technology; auditing; and appropriate attitudinal change to ensure improved patient service and research outcomes and attainment of the highest degree of satisfaction by stakeholders. |
| Dentist | Dentists diagnose, treat and prevent diseases, injuries and abnormalities of the teeth, mouth, jaws and associated tissues by applying the principles and procedures of modern dentistry. They use a broad range of specialized diagnostic, surgical and other techniques to promote and restore oral health. |
| Dental assistants and therapists | Dental assistants and therapists provide basic dental care services for the prevention and treatment of diseases and disorders of the teeth and mouth, according to care plans and procedures established by a dentist or other oral health professional. Under this dental assistants and therapists, dental hygienist and dental nurse will be covered. |
| Dental aids | Key dental personnel who are involved in infection control, organization of the dental surgery, prepare, mix and handle dental materials, provide chair side support to the operator during treatment. Under this dental surgery assistants and dental nurses (non-clinical) will be covered. |
| Dental Prosthetic/Technician | Dental prosthetic technicians design, fit, service and repair dental devices and appliances following prescriptions or instructions established by a health professional. They may service a wide range of support instruments to correct dental problems, such as dentures, and dental crowns and bridges. This will include clinical and non-clinical technicians. |
| Foreign-trained dentists | Health workers who have obtained their first medical qualification (degree) in another country and are entitled to practise in the receiving country |
| Inter-professional education system | Inter-professional education occurs when two or more health professionals learn about, from and with each other to enable effective collaboration and improve health outcomes. Professional is an  all-encompassing term that includes individuals with the knowledge and/or skills to contribute to the physical, mental and social well-being of a community. |
| License, certificate | The license or certification is the permission to practise in the appropriate field of health, issued by a legitimate regulatory body within the profession |
| Skill mix | A relatively broad term that can refer to the mix of staff in the workforce or the demarcation of roles and activities among different categories of staff |

If you need help completing the survey at any point please contact: makinoy@who.int

End of Block: Introduction

Start of Block: Section 1. You and Your Country

**Module 1: General information**

**1.1   Country**

▼ Afghanistan (1) ... Zimbabwe (194)

**1.2  Details of personnel completing the survey** (please state Not Available (NA) where appropriate)

|  | Responsible officer name (1) | Position (2) | Institution (3) | Email address (4) |
| --- | --- | --- | --- | --- |
| All modules (1) |  |  |  |  |
| Module 1 (6) |  |  |  |  |
| Module 2 (7) |  |  |  |  |
| Module 3 (8) |  |  |  |  |
| Module 4 (9) |  |  |  |  |

End of Block: Section 1. You and Your Country

Start of Block: Section 2. Oral Health Workforce Overview

**Module 2: Oral Health Workforce: capacity, capability and governance**
 
Please fill in all of the blanks. If you do not know answer, or not available for your country please add Don't Know (DK) or Not Available (NA).

**2.1   Briefly, please list the types of oral health workforce  (i.e. dentists, dental nurses, hygienists, therapists, technicians, etc) working in your country?**

________________________________________________________________

**2.2 Which organization, or organizations, hold dental workforce data in your country?**

________________________________________________________________

**2.3 Which oral health workforce are working in your country, how many of them are there currently, and are they regulated?**  Taking each one in turn, please provide the following information on numbers. *(Please complete all relevant boxes and answer as best you can)*

**2.3.1 Which oral health workforce are working in your country and how many of them are there currently?**

1. Dentists

|  | Size of workforce | | |
| --- | --- | --- | --- |
|  | Number (1) | Is this an estimate (state YES or NO) (2) | Year of data (3) |
| Dentists (1) |  |  |  |

Dentists diagnose, treat and prevent diseases, injuries and abnormalities of the teeth, mouth, jaws and associated tissues by applying the principles and procedures of modern dentistry. They use a broad range of specialized diagnostic, surgical and other techniques to promote and restore oral health.

2. Dental assistants and therapists

|  | Size of workforce | | |
| --- | --- | --- | --- |
|  | Number (1) | Is this an estimate (state YES or NO) (2) | Year of data (3) |
| 2a. Dental therapists (2) |  |  |  |
| 2b. Dental hygienists (3) |  |  |  |
| 2c. Dental assistants (11) |  |  |  |
| 2d. Dental nurses (4) |  |  |  |

Q46 Dental assistants and therapists provide basic dental care services for the prevention and treatment of diseases and disorders of the teeth and mouth, according to care plans and procedures established by a dentist or other oral health professional. Under this dental assistants and therapists, dental hygienists and dental nurses (clinical) will be covered.

3. Dental prosthetic technicians

|  | Size of workforce | | |
| --- | --- | --- | --- |
|  | Number (1) | Is this an estimate (state YES or NO) (2) | Year of data (3) |
| Dental prosthetic technicians (6) |  |  |  |

Dental prosthetic technicians design, fit, service and repair dental devices and appliances following prescriptions or instructions established by a health professional. They may service a wide range of support instruments to correct dental problems, such as dentures, and dental crowns and bridges. This will include clinical and non-clinical technicians.

4. Dental aides

|  | Size of workforce | | |
| --- | --- | --- | --- |
|  | Number (1) | Is this an estimate (state YES or NO) (2) | Year of data (3) |
| Dental aides (12) |  |  |  |

Dental aides are key dental personnel who are involved in infection control, organization of the dental surgery, prepare, mix and handle dental materials, provide chair side support to the operator during treatment. Under this dental aides, dental surgery assistants and dental nurses (non-clinical) will be covered.

5. Other (if your country has other oral health workforce, please specify here. If not, please add NA (not available))

|  | Size of workforce | | |
| --- | --- | --- | --- |
|  | Number (1) | Is this an estimate (state YES or NO) (2) | Year of data (3) |
| Other (11) |  |  |  |

**2.3.2 If permission (license, certificate, registration) is required to practice, please select appropriate responsible bodies from the drop down list based on your country context.**

|  | License/ certificate | Registration |
| --- | --- | --- |
|  |  |  |
| 1. Dentists (1) | ▼ Government ex. MOH (1 ... Don't Know (5) | ▼ Government ex. MOH (1 ... Don't Know (5) |
| 2a. Dental therapists (2) | ▼ Government ex. MOH (1 ... Don't Know (5) | ▼ Government ex. MOH (1 ... Don't Know (5) |
| 2b. Dental hygienists (3) | ▼ Government ex. MOH (1 ... Don't Know (5) | ▼ Government ex. MOH (1 ... Don't Know (5) |
| 2c. Dental assistants (8) | ▼ Government ex. MOH (1 ... Don't Know (5) | ▼ Government ex. MOH (1 ... Don't Know (5) |
| 2d. Dental nurses (4) | ▼ Government ex. MOH (1 ... Don't Know (5) | ▼ Government ex. MOH (1 ... Don't Know (5) |
| 3. Dental prosthetist technicians (5) | ▼ Government ex. MOH (1 ... Don't Know (5) | ▼ Government ex. MOH (1 ... Don't Know (5) |
| 4. Dental aides (10) | ▼ Government ex. MOH (1 ... Don't Know (5) | ▼ Government ex. MOH (1 ... Don't Know (5) |
| 5. Other (7) | ▼ Government ex. MOH (1 ... Don't Know (5) | ▼ Government ex. MOH (1 ... Don't Know (5) |

**2.3.3 Are community health workers involved in oral health activities?**

▼ Don't Know (1) ... No (2)

**2.3.4 If yes, what percentages of community health workers are involved in the oral health activities?** (If no, then select don't know)

▼ Don't know (1) ... 99 (100)

**2.4 What proportion of the oral health workforce as clinical practitioners are in respective sectors?** Percentage of (active) health workers employed in facilities by type of ownership (public, private) *[please ensure all boxes have been completed using the botton control bar]*

|  | Public | | Private | | Public/ private mix | | Other (e.g non-profit organizations such as NGOs, church etc) | | Inactive (e.g unemployed, maternity leave, sabbatical year etc) | |
| --- | --- | --- | --- | --- | --- | --- | --- | --- | --- | --- |
|  | % (1) | Number (2) | % (1) | Number (2) | % (1) | Number (2) | % (1) | Number (2) | % (1) | Number (2) |
| 1. Dentists (1) |  |  |  |  |  |  |  |  |  |  |
| 2a. Dental therapists (2) |  |  |  |  |  |  |  |  |  |  |
| 2b. Dental hygienists (3) |  |  |  |  |  |  |  |  |  |  |
| 2c. Dental assistants (4) |  |  |  |  |  |  |  |  |  |  |
| 2d. Dental nurses (5) |  |  |  |  |  |  |  |  |  |  |
| 3. Dental prosthetic technicians (6) |  |  |  |  |  |  |  |  |  |  |
| 4. Dental aides (10) |  |  |  |  |  |  |  |  |  |  |
| 5. Community health workers (7) |  |  |  |  |  |  |  |  |  |  |
| 6. Other (8) |  |  |  |  |  |  |  |  |  |  |

**2.5 What is the gender distribution of ACTIVE dentists?**

- Majority male (1)
- Majority female (2)
- 50/50 (3)
- Don't know (4)

**2.6 What is the gender distribution of ACTIVE dental assistants and therapists?**

- Majority male (1)
- Majority female (2)
- 50/50 (3)
- Don't know (4)

2.7 What is the age distribution of ACTIVE dentists? (answer in percentage)

|  |  |
| --- | --- |
| a. Under 25 years (1) | ▼ Don't Know (1) ... 100 (101) |
| b. 25-34 years (2) | ▼ Don't Know (1) ... 100 (101) |
| c. 35-44 years (3) | ▼ Don't Know (1) ... 100 (101) |
| d. 45-54 years (4) | ▼ Don't Know (1) ... 100 (101) |
| e. 55-64 years (5) | ▼ Don't Know (1) ... 100 (101) |
| f. 65 years and above (6) | ▼ Don't Know (1) ... 100 (101) |

2.8 What is the age distribution of ACTIVE dental assistants and therapists? (answer in percentage)

|  |  |
| --- | --- |
| a. Under 25 years (1) | ▼ Don't Know (1) ... 100 (101) |
| b. 25-34 years (2) | ▼ Don't Know (1) ... 100 (101) |
| c. 35-44 years (3) | ▼ Don't Know (1) ... 100 (101) |
| d. 45-54 years (4) | ▼ Don't Know (1) ... 100 (101) |
| e. 55-64 years (5) | ▼ Don't Know (1) ... 100 (101) |
| f. 65 years and above (6) | ▼ Don't Know (1) ... 100 (101) |

2.9 What is the proportion of ACTIVE foreign-trained dentists work in your country? (answer in percentage)

▼ Don't know (1) ... 99 (100)

**2.10 Does your country have dental specialties?**

▼ Yes (4) ... No (5)

**2.10.1 If yes, please list the types of dental specialists that practice in your country.**

________________________________________________________________

End of Block: Section 2. Oral Health Workforce Overview

Start of Block: Section 3. Education and training of the oral health workforce

**Module 3: Education and training of the oral health workforce**
 
Please fill in all of the blanks. If you do not know answer, or not available for your country please add Don't Know (DK) or Not Available (NA).

A. Dental Schools

|  | Number (1) | Length of course (years) (3) | Comments (4) |
| --- | --- | --- | --- |
| 3.1 How many dental schools do you have in your country in *total*? (1) |  |  |  |
| 3.2 How many *public*  dental schools in your country? (2) |  |  |  |
| 3.3 How many *private*  dental schools in your country? (3) |  |  |  |

B. Dental Assistants and Therapists Schools (dental hygienists and clinical dental nurses are included in this group)

|  | Number (1) | Length of course (years) (3) | Comments (4) |
| --- | --- | --- | --- |
| 3.4 How many schools for dental assistants and therapists are there in your country in *total*? (1) |  |  |  |
| 3.5 How many *public schools* for dental assistants and therapists in your country? (2) |  |  |  |
| 3.6 How many *private schools* for dental assistants and therapists in your country? (3) |  |  |  |

C. New graduates

|  | Total number (3) | Graduates starting practice within one year (%) (4) |
| --- | --- | --- |
| 3.7 How many dentist graduated last year from *public* dental schools? (1) |  |  |
| 3.8 How many dentist graduates last year from *private* dental schools? (2) |  |  |
| 3.9 How many dental assistants and therapists graduated last year from *public* schools? (3) |  |  |
| 3.10 How many dental assistants and therapists graduated last year from *private* schools? (4) |  |  |

**Dentists specific questions**

D. Dentists’ accreditation system and system for foreign-trained dentists

|  | Yes (2) | No (3) | Don't know (4) |
| --- | --- | --- | --- |
| 3.11 Does your country have accreditation mechanisms for dental school education? (1) |  |  |  |
| 3.12 Does your country have formal mechanism for accreditation of diplomas/ degrees obtained abroad? (2) |  |  |  |

E. Inter-professional education system

|  | Yes (1) | No (2) | Don't know (3) |
| --- | --- | --- | --- |
| 3.13 Does your country have national and/or subnational standards for inter-professional education? (1) |  |  |  |
| 3.14 Does your country have a formal mechanism for the recognition of inter-professional education? (2) |  |  |  |

F. Existence of national systems for continuing professional development

|  | Yes (1) | No (2) |
| --- | --- | --- |
| 3.15 Do you have national systems for continuing professional development? (1) |  |  |
| 3.15.1 If yes, is it mandatory to participate to maintain licensing? If no, please proceed to the next module. (2) |  |  |

End of Block: Section 3. Education and training of the oral health workforce

Start of Block: Section 4. Better use of the oral health workforce

**Module 4: Better use of the oral health workforce**

**4.1  Challenges related to oral health workforce in your country. To what extent do you agree with the following statements** (strongly disagree [1] to strongly agree [10])?

|  | Strongly disagree | Strongly agree |
| --- | --- | --- |

|  | 1 | 2 | 3 | 4 | 5 | 6 | 7 | 8 | 9 | 10 |
| --- | --- | --- | --- | --- | --- | --- | --- | --- | --- | --- |

| a. Training too many dentists () | 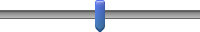 |
| --- | --- |
| b. Training too few dentists () | 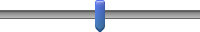 |
| c. Migration of dentists into the country () | 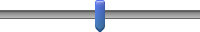 |
| d. Migration of dentists out of the country () | 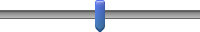 |
| e. Training too many dental assistants and therapists () | 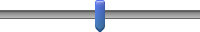 |
| f. Training too few dental assistants and therapists () | 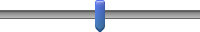 |
| g. Limited jobs (vacancies) for dentists () | 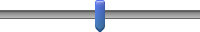 |
| h. Limited jobs (vacancies) for dental assistants and therapists () | 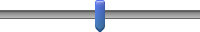 |
| i. Lack of diversity (skill mix) in the dental team () | 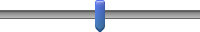 |
| j. Mal-distribution of the workforce (ex. Urban, Rural) () | 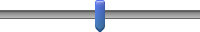 |
| k. Poor quality dental care () | 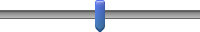 |
| l. Lack of continuing professional development opportunities () | 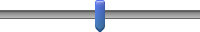 |
| m. Existence of unregistered providers of dentistry, e.g. quacks and traditional healers () | 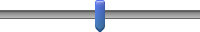 |
| n. Lack of financial support for oral health workforce training institutions () | 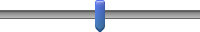 |
| o. Lack of workforce data for planning () | 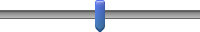 |
| p. Oral health is considered low priority () | 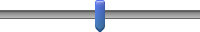 |
| q. Other (please specify) () | 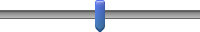 |

**4.2 Solutions related to oral health workforce in your country.**To what extent do you agree with the following statements (strongly disagree [1] to strongly agree [10])?

|  | Strongly disagree | Strongly agree |
| --- | --- | --- |

|  | 1 | 2 | 3 | 4 | 5 | 6 | 7 | 8 | 9 | 10 |
| --- | --- | --- | --- | --- | --- | --- | --- | --- | --- | --- |

| a. Training more dentists () | 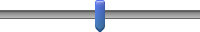 |
| --- | --- |
| b. Training fewer dentists () | 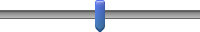 |
| c. Reducing migration of dentists out of the country () | 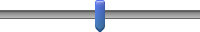 |
| d. Reducing migration of dentists into the country () | 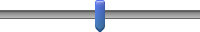 |
| e. Training fewer dental assistants and therapists () | 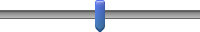 |
| f. Training more dental assistants and therapists () | 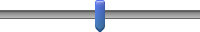 |
| g. Creating jobs for dentists () | 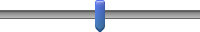 |
| h. Creating jobs for dental assistants and therapists () | 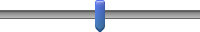 |
| i. Workforce incentives to work in underserved areas () | 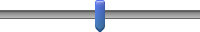 |
| j. Creating diversity (skill mix) in the oral health workforce () | 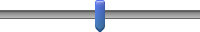 |
| k. Strengthened quality of dental care () | 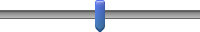 |
| l. Regulating unregistered providers of dentistry, e.g. quacks and traditional healers () | 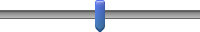 |
| m.Financial support for dental personnel education () | 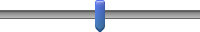 |
| n. Improve health workforce data for planning () | 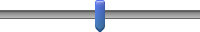 |
| o. Regulation of dental education () | 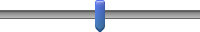 |
| p. Regulation of national systems for continuing professional development () | 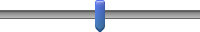 |
| q. Strengthen oral health policy () | 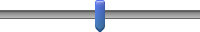 |
| r. Other (please specify) () | 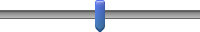 |

**4.3 How would you like to see dentistry changing in your country? E.g skill mix, inter-professional working**

________________________________________________________________

End of Block: Section 4. Better use of the oral health workforce

Start of Block: Conclusion

THANK YOU FOR TAKING THE TIME TO ANSWER THIS QUESTIONNAIRE   As far as possible, in order to validate responses, documentation will be requested for affirmative responses throughout the questionnaire. Please make every effect to provide electronic copies of the requested documentation by email: varenneb@who.int and makinoy@who.int If you are unable to provide the electronic copies through the email, please contact varenneb@who.int and makinoy@who.int for an alternative means to submit documentation.

**Going to the next page will submit the survey and you will not be able to return to previous questions after your submission.**

End of Block: Conclusion
