# Supplementary material for: Health workforce for oral health inequity: Opportunity for action
Source: PLoS One. 2024 Jun 13;19(6):e0292549. doi: 10.1371/journal.pone.0292549 (PMC11175420; doi:10.1371/journal.pone.0292549)
Supplement: S1 Table — (DOCX) [file pone.0292549.s003.docx]

**Supplementary Information 3: Global data availability per country and professional category**

Available: **🗸**

Not available: 🗴

| **Countries** | **Dentists** | **DATs (Assistants and Therapists)** | **DPTs (Prosthetics Technicians)** |
| --- | --- | --- | --- |
| Afghanistan | **🗸** | **🗸** | **🗸** |
| Albania | **🗸** | **🗸** | **🗸** |
| Algeria | **🗸** | **🗸** | **🗸** |
| Andorra | **🗸** | **🗴** | **🗴** |
| Angola | **🗸** | **🗸** | **🗴** |
| Antigua and Barbuda | **🗸** | **🗸** | **🗴** |
| Argentina | **🗸** | **🗴** | **🗸** |
| Armenia | **🗸** | **🗴** | **🗴** |
| Australia | **🗸** | **🗸** | **🗸** |
| Austria | **🗸** | **🗸** | **🗸** |
| Azerbaijan | **🗸** | **🗴** | **🗴** |
| Bahamas | **🗸** | **🗸** | **🗸** |
| Bahrain | **🗸** | **🗸** | **🗸** |
| Bangladesh | **🗸** | **🗴** | **🗸** |
| Barbados | **🗸** | **🗸** | **🗸** |
| Belarus | **🗸** | **🗸** | **🗸** |
| Belgium | **🗸** | **🗴** | **🗴** |
| Belize | **🗸** | **🗸** | **🗸** |
| Benin | **🗸** | **🗸** | **🗸** |
| Bhutan | **🗸** | **🗸** | **🗸** |
| Bolivia (Plurinational State of) | **🗸** | **🗸** | **🗴** |
| Bosnia and Herzegovina | **🗸** | **🗴** | **🗴** |
| Botswana | **🗸** | **🗸** | **🗸** |
| Brazil | **🗸** | **🗸** | **🗸** |
| Brunei Darussalam | **🗸** | **🗸** | **🗸** |
| Bulgaria | **🗸** | **🗸** | **🗸** |
| Burkina Faso | **🗸** | **🗸** | **🗸** |
| Burundi | **🗸** | **🗴** | **🗴** |
| Cabo Verde | **🗸** | **🗸** | **🗸** |
| Cambodia | **🗸** | **🗸** | **🗴** |
| Cameroon | **🗸** | **🗴** | **🗴** |
| Canada | **🗸** | **🗸** | **🗸** |
| Central African Republic | **🗸** | **🗴** | **🗸** |
| Chad | **🗸** | **🗸** | **🗸** |
| Chile | **🗸** | **🗴** | **🗴** |
| China | **🗸** | **🗴** | **🗴** |
| Colombia | **🗸** | **🗴** | **🗴** |
| Comoros | **🗸** | **🗸** | **🗸** |
| Congo | **🗸** | **🗸** | **🗸** |
| Cook Islands | **🗸** | **🗸** | **🗸** |
| Costa Rica | **🗸** | **🗸** | **🗸** |
| Cote d'Ivoire | **🗸** | **🗸** | **🗸** |
| Croatia | **🗸** | **🗸** | **🗸** |
| Cuba | **🗸** | **🗸** | **🗸** |
| Cyprus | **🗸** | **🗸** | **🗸** |
| Czechia | **🗸** | **🗸** | **🗸** |
| Democratic People's Republic of Korea | **🗸** | **🗴** | **🗸** |
| Democratic Republic of the Congo | **🗸** | **🗴** | **🗸** |
| Denmark | **🗸** | **🗴** | **🗴** |
| Djibouti | **🗸** | **🗴** | **🗴** |
| Dominica | **🗸** | **🗴** | **🗴** |
| Dominican Republic | **🗸** | **🗸** | **🗸** |
| Ecuador | **🗸** | **🗸** | **🗴** |
| Egypt | **🗸** | **🗴** | **🗸** |
| El Salvador | **🗸** | **🗸** | **🗴** |
| Equatorial Guinea | **🗸** | **🗸** | **🗸** |
| Eritrea | **🗸** | **🗸** | **🗴** |
| Estonia | **🗸** | **🗸** | **🗸** |
| Eswatini | **🗸** | **🗸** | **🗸** |
| Ethiopia | **🗸** | **🗸** | **🗸** |
| Fiji | **🗸** | **🗸** | **🗸** |
| Finland | **🗸** | **🗸** | **🗸** |
| France | **🗸** | **🗴** | **🗴** |
| Gabon | **🗸** | **🗸** | **🗸** |
| Gambia | **🗸** | **🗸** | **🗸** |
| Georgia | **🗸** | **🗸** | **🗸** |
| Germany | **🗸** | **🗸** | **🗸** |
| Ghana | **🗸** | **🗸** | **🗸** |
| Greece | **🗸** | **🗸** | **🗴** |
| Grenada | **🗸** | **🗴** | **🗸** |
| Guatemala | **🗸** | **🗴** | **🗴** |
| Guinea | **🗸** | **🗴** | **🗸** |
| Guinea-Bissau | **🗸** | **🗸** | **🗸** |
| Guyana | **🗸** | **🗸** | **🗸** |
| Haiti | **🗸** | **🗸** | **🗸** |
| Honduras | **🗸** | **🗴** | **🗸** |
| Hungary | **🗸** | **🗸** | **🗸** |
| Iceland | **🗸** | **🗸** | **🗸** |
| India | **🗸** | **🗸** | **🗸** |
| Indonesia | **🗸** | **🗸** | **🗸** |
| Iran (Islamic Republic of) | **🗸** | **🗸** | **🗸** |
| Iraq | **🗸** | **🗸** | **🗸** |
| Ireland | **🗸** | **🗴** | **🗴** |
| Israel | **🗸** | **🗴** | **🗴** |
| Italy | **🗸** | **🗴** | **🗴** |
| Jamaica | **🗸** | **🗸** | **🗸** |
| Japan | **🗸** | **🗸** | **🗸** |
| Jordan | **🗸** | **🗸** | **🗸** |
| Kazakhstan | **🗸** | **🗴** | **🗴** |
| Kenya | **🗸** | **🗸** | **🗸** |
| Kiribati | **🗸** | **🗴** | **🗸** |
| Kuwait | **🗸** | **🗴** | **🗴** |
| Kyrgyzstan | **🗸** | **🗸** | **🗸** |
| Lao People's Democratic Republic | **🗸** | **🗸** | **🗴** |
| Latvia | **🗸** | **🗸** | **🗸** |
| Lebanon | **🗸** | **🗴** | **🗴** |
| Lesotho | **🗸** | **🗸** | **🗸** |
| Liberia | **🗸** | **🗸** | **🗸** |
| Libya | **🗸** | **🗴** | **🗴** |
| Lithuania | **🗸** | **🗴** | **🗴** |
| Luxembourg | **🗸** | **🗴** | **🗸** |
| Madagascar | **🗸** | **🗸** | **🗸** |
| Malawi | **🗸** | **🗸** | **🗴** |
| Malaysia | **🗸** | **🗸** | **🗸** |
| Maldives | **🗸** | **🗸** | **🗸** |
| Mali | **🗸** | **🗸** | **🗸** |
| Malta | **🗸** | **🗸** | **🗸** |
| Marshall Islands | **🗸** | **🗸** | **🗸** |
| Mauritania | **🗸** | **🗸** | **🗸** |
| Mauritius | **🗸** | **🗸** | **🗸** |
| Mexico | **🗸** | **🗸** | **🗸** |
| Micronesia (Federated States of) | **🗸** | **🗴** | **🗸** |
| Monaco | **🗸** | **🗴** | **🗴** |
| Mongolia | **🗸** | **🗸** | **🗴** |
| Montenegro | **🗸** | **🗸** | **🗸** |
| Morocco | **🗸** | **🗴** | **🗴** |
| Mozambique | **🗸** | **🗸** | **🗸** |
| Myanmar | **🗸** | **🗸** | **🗸** |
| Namibia | **🗸** | **🗸** | **🗸** |
| Nauru | **🗸** | **🗸** | **🗸** |
| Nepal | **🗸** | **🗸** | **🗸** |
| Netherlands | **🗸** | **🗸** | **🗸** |
| New Zealand | **🗸** | **🗸** | **🗸** |
| Nicaragua | **🗸** | **🗸** | **🗴** |
| Niger | **🗸** | **🗴** | **🗸** |
| Nigeria | **🗸** | **🗸** | **🗸** |
| Niue | **🗸** | **🗴** | **🗸** |
| North Macedonia | **🗸** | **🗴** | **🗴** |
| Norway | **🗸** | **🗸** | **🗸** |
| Oman | **🗸** | **🗸** | **🗴** |
| Pakistan | **🗸** | **🗸** | **🗸** |
| Palau | **🗸** | **🗸** | **🗸** |
| Panama | **🗸** | **🗴** | **🗴** |
| Papua New Guinea | **🗸** | **🗸** | **🗸** |
| Paraguay | **🗸** | **🗸** | **🗸** |
| Peru | **🗸** | **🗸** | **🗴** |
| Philippines | **🗸** | **🗸** | **🗸** |
| Poland | **🗸** | **🗴** | **🗴** |
| Portugal | **🗸** | **🗸** | **🗸** |
| Qatar | **🗸** | **🗸** | **🗸** |
| Republic of Korea | **🗸** | **🗸** | **🗸** |
| Republic of Moldova | **🗸** | **🗴** | **🗴** |
| Romania | **🗸** | **🗸** | **🗴** |
| Russian Federation | **🗸** | **🗴** | **🗴** |
| Rwanda | **🗸** | **🗸** | **🗸** |
| Saint Kitts and Nevis | **🗸** | **🗴** | **🗴** |
| Saint Lucia | **🗸** | **🗴** | **🗴** |
| Saint Vincent and the Grenadines | **🗸** | **🗴** | **🗴** |
| Samoa | **🗸** | **🗸** | **🗸** |
| San Marino | **🗸** | **🗸** | **🗸** |
| Sao Tome and Principe | **🗸** | **🗸** | **🗸** |
| Saudi Arabia | **🗸** | **🗴** | **🗸** |
| Senegal | **🗸** | **🗸** | **🗸** |
| Serbia | **🗸** | **🗸** | **🗸** |
| Seychelles | **🗸** | **🗸** | **🗸** |
| Sierra Leone | **🗸** | **🗸** | **🗸** |
| Singapore | **🗸** | **🗸** | **🗴** |
| Slovakia | **🗸** | **🗸** | **🗸** |
| Slovenia | **🗸** | **🗸** | **🗸** |
| Solomon Islands | **🗸** | **🗸** | **🗸** |
| Somalia | **🗴** | **🗴** | **🗴** |
| South Africa | **🗸** | **🗸** | **🗸** |
| South Sudan | **🗸** | **🗸** | **🗸** |
| Spain | **🗸** | **🗸** | **🗸** |
| Sri Lanka | **🗸** | **🗸** | **🗸** |
| Sudan | **🗸** | **🗸** | **🗸** |
| Suriname | **🗸** | **🗴** | **🗴** |
| Sweden | **🗸** | **🗸** | **🗴** |
| Switzerland | **🗸** | **🗴** | **🗴** |
| Syrian Arab Republic | **🗸** | **🗴** | **🗴** |
| Tajikistan | **🗸** | **🗴** | **🗴** |
| Thailand | **🗸** | **🗸** | **🗸** |
| Timor-Leste | **🗸** | **🗸** | **🗸** |
| Togo | **🗸** | **🗸** | **🗸** |
| Tonga | **🗸** | **🗸** | **🗸** |
| Trinidad and Tobago | **🗸** | **🗸** | **🗴** |
| Tunisia | **🗸** | **🗴** | **🗸** |
| Turkey | **🗸** | **🗴** | **🗴** |
| Turkmenistan | **🗸** | **🗴** | **🗴** |
| Tuvalu | **🗸** | **🗴** | **🗸** |
| Uganda | **🗸** | **🗸** | **🗸** |
| Ukraine | **🗸** | **🗴** | **🗴** |
| United Arab Emirates | **🗸** | **🗴** | **🗴** |
| United Kingdom | **🗸** | **🗴** | **🗴** |
| United Republic of Tanzania | **🗸** | **🗸** | **🗸** |
| United States of America | **🗸** | **🗸** | **🗴** |
| Uruguay | **🗸** | **🗸** | **🗴** |
| Uzbekistan | **🗸** | **🗴** | **🗴** |
| Vanuatu | **🗸** | **🗸** | **🗴** |
| Venezuela (Bolivarian Republic of) | **🗸** | **🗴** | **🗴** |
| Viet Nam | **🗴** | **🗴** | **🗴** |
| Yemen | **🗸** | **🗸** | **🗴** |
| Zambia | **🗸** | **🗸** | **🗸** |
| Zimbabwe | **🗸** | **🗸** | **🗸** |

Source: The workforce data are based on the latest available data in the NHWA data platform as of 31 March 2021, apart from the data for 2019, which a combination of the latest available data from the NHWA data platform and King’s College London survey was used.
