# Supplementary material for: Health workforce for oral health inequity: Opportunity for action
Source: PLoS One. 2024 Jun 13;19(6):e0292549. doi: 10.1371/journal.pone.0292549 (PMC11175420; doi:10.1371/journal.pone.0292549)
Supplement: S2 Table — (DOCX) [file pone.0292549.s004.docx]

**S4: Global dataset by country: density of dentists per 10,000 population globally, 2000-2019**

| Country | Year | | | | | | | | | | | | | | | | | | | |
| --- | --- | --- | --- | --- | --- | --- | --- | --- | --- | --- | --- | --- | --- | --- | --- | --- | --- | --- | --- | --- |
|  | 2000 | 2001 | 2002 | 2003 | 2004 | 2005 | 2006 | 2007 | 2008 | 2009 | 2010 | 2011 | 2012 | 2013 | 2014 | 2015 | 2016 | 2017 | 2018 | 2019 |
| Afghanistan |  |  |  |  |  |  | 0.127 | 0.115 | 0.138 | 0.138 | 0.138 | 0.138 | 0.138 | 0.138 | 0.033 | 0.036 | 0.034 | 0.034 | 0.034 | 0.709 |
| Albania | 4.656 | 4.873 | 4.293 | 4.33 | 4.33 | 4.33 | 3.379 | 3.379 | 3.379 | 3.379 | 3.379 | 3.379 | 3.379 | 3.379 | 3.379 | 3.379 | 3.379 | 3.379 | 10.313 | 10.313 |
| Algeria |  |  | 2.782 | 2.782 | 2.782 | 2.722 | 2.722 | 3.109 | 3.109 | 3.109 | 3.109 | 3.109 | 3.109 | 3.109 | 3.109 | 3.109 | 3.109 | 3.446 | 3.656 | 3.656 |
| Andorra | 6.576 | 6.237 | 5.996 | 6.013 | 5.902 | 5.832 | 5.679 | 5.684 | 5.684 | 6.038 | 6.038 | 6.038 | 6.038 | 6.038 | 6.038 | 8.206 | 8.206 | 8.206 | 8.206 | 8.206 |
| Angola |  |  |  |  | 0.118 | 0.118 | 0.118 | 0.118 | 0.118 | 0.118 | 0.118 | 0.118 | 0.118 | 0.118 | 0.118 | 0.118 | 0.118 | 0.118 | 0.011 | 0.011 |
| Antigua and Barbuda |  |  |  |  |  |  |  |  |  |  |  |  |  |  | 0.432 | 0.432 | 0.432 | 0.432 | 0.432 | 0.432 |
| Argentina |  | 9.643 | 9.643 | 9.643 | 9.247 | 9.247 | 9.247 | 9.247 | 9.247 | 9.247 | 9.247 | 9.247 | 9.247 | 9.247 | 9.247 | 9.247 | 9.247 | 9.247 | 15.347 | 15.347 |
| Armenia | 2.417 | 1.691 | 1.931 | 2.084 | 2.353 | 3.217 | 3.194 | 3.441 | 4.117 | 4.311 | 4.369 | 4.509 | 4.473 | 4.193 | 3.907 | 3.692 | 3.692 | 5.562 | 5.562 | 5.562 |
| Australia | 0 | 4.272 | 4.272 | 4.272 | 4.272 | 4.992 | 4.872 | 4.872 | 4.872 | 5.266 | 5.179 | 5.352 | 5.574 | 5.611 | 5.709 | 5.784 | 5.855 | 5.947 | 5.947 | 5.947 |
| Austria | 4.613 | 4.79 | 4.863 | 4.938 | 5.006 | 5.127 | 5.391 | 5.401 | 5.447 | 5.517 | 5.571 | 5.611 | 5.642 | 5.672 | 5.679 | 5.653 | 5.663 | 5.679 | 5.679 | 5.679 |
| Azerbaijan | 2.75 | 2.751 | 2.809 | 2.721 | 2.747 | 2.803 | 2.817 | 2.891 | 2.818 | 2.753 | 2.731 | 2.625 | 2.656 | 2.613 | 2.736 | 2.736 | 2.736 | 2.736 | 2.736 | 2.736 |
| Bahamas |  |  |  |  |  |  |  |  | 2.299 | 2.299 | 2.299 | 2.336 | 2.336 | 2.336 | 2.336 | 2.336 | 2.336 | 2.593 | 2.593 | 2.593 |
| Bahrain |  |  |  |  | 2.169 | 1.102 | 1.075 | 1.075 | 1.068 | 0.996 | 0.983 | 0.994 | 1.069 | 1.019 | 0.98 | 0.977 | 0.977 | 0.977 | 0.977 | 0.977 |
| Bangladesh |  |  | 0.142 | 0.142 | 0.142 | 0.169 | 0.169 | 0.144 | 0.234 | 0.263 | 0.294 | 0.323 | 0.319 | 0.395 | 0.412 | 0.417 | 0.444 | 0.511 | 0.595 | 0.589 |
| Barbados |  | 2.202 | 2.202 | 2.202 | 2.202 | 3.004 | 3.004 | 3.004 | 3.004 | 3.004 | 3.004 | 3.004 | 3.004 | 3.004 | 3.004 | 3.004 | 3.004 | 3.074 | 3.074 | 3.074 |
| Belarus | 4.543 | 4.477 | 4.488 | 4.458 | 4.634 | 4.764 | 4.883 | 5.046 | 5.411 | 5.493 | 5.4 | 5.441 | 5.516 | 5.514 | 5.74 | 5.923 | 5.923 | 5.923 | 6.228 | 6.228 |
| Belgium | 7.449 | 7.443 | 7.414 | 7.371 | 7.359 | 7.295 | 7.296 | 7.176 | 7.109 | 7.049 | 7.016 | 7.061 | 7.099 | 7.141 | 7.226 | 7.345 | 7.467 | 7.457 | 10.474 | 10.474 |
| Belize | 1.294 | 1.294 | 1.294 | 1.294 | 1.294 | 1.294 | 1.294 | 1.294 | 1.294 | 0.381 | 0.372 | 0.372 | 0.372 | 0.372 | 0.372 | 0.372 | 0.372 | 1.543 | 1.488 | 1.383 |
| Benin |  |  |  |  | 0.014 | 0.014 | 0.014 | 0.014 | 0.043 | 0.043 | 0.043 | 0.043 | 0.043 | 0.043 | 0.043 | 0.043 | 0.008 | 0.065 | 0.01 | 0.01 |
| Bhutan |  |  |  |  | 0.157 | 0.157 | 0.157 | 0.105 | 0.105 | 0.105 | 0.105 | 0.105 | 0.105 | 0.105 | 0.105 | 0.105 | 0.105 | 0.899 | 0.795 | 0.773 |
| Bolivia (Plurinational State of) |  |  |  |  |  |  |  | 0.626 | 0.626 | 0.626 | 0.971 | 1.106 | 1.106 | 1.106 | 1.106 | 1.106 | 2.201 | 1.799 | 1.799 | 1.799 |
| Bosnia and Herzegovina | 1.933 | 1.808 | 1.825 | 1.223 | 1.918 | 1.917 | 2.045 | 1.988 | 1.795 | 1.82 | 2.151 | 2.207 | 2.427 | 2.284 | 2.369 | 2.4 | 2.4 | 2.4 | 2.4 | 2.4 |
| Botswana |  |  |  |  | 0.215 | 0.215 | 0.215 | 0.171 | 0.094 | 0.282 | 0.277 | 0.273 | 0.27 | 0.27 | 0.27 | 0.27 | 0.421 | 0.421 | 0.421 | 0.747 |
| Brazil | 8.777 | 8.715 | 9.09 | 9.403 | 9.675 | 11.17 | 11.544 | 11.562 | 11.828 | 11.935 | 11.395 | 11.395 | 11.395 | 11.395 | 11.395 | 11.395 | 11.395 | 12.454 | 6.4 | 6.4 |
| Brunei Darussalam | 1.441 | 1.441 | 2.019 | 2.019 | 1.892 | 1.999 | 2.026 | 2.16 | 2.161 | 1.875 | 2.213 | 2.413 | 2.356 | 2.356 | 2.356 | 2.266 | 2.266 | 2.266 | 2.266 | 2.539 |
| Bulgaria | 8.475 | 8.173 | 7.811 | 8.297 | 8.403 | 8.477 | 8.566 | 8.527 | 8.392 | 8.736 | 8.605 | 9.056 | 9.204 | 10.004 | 9.735 | 10.482 | 10.482 | 10.482 | 10.267 | 14 |
| Burkina Faso |  |  |  |  | 0.045 | 0.045 | 0.045 | 0.045 | 0.045 | 0.045 | 0.021 | 0.021 | 0.023 | 0.023 | 0.023 | 0.023 | 0.023 | 0.042 | 0.046 | 0.046 |
| Burundi |  |  |  |  | 0.02 | 0.02 | 0.02 | 0.02 | 0.02 | 0.02 | 0.002 | 0.002 | 0.002 | 0.004 | 0.004 | 0.004 | 0.004 | 0.004 | 0.004 | 0.004 |
| Cabo Verde |  |  |  |  |  |  | 0.064 | 0.063 | 0.062 | 0.062 | 0.081 | 0.06 | 0.06 | 0.06 | 0.06 | 0.06 | 0.075 | 0.075 | 0.075 | 2.182 |
| Cambodia | 0.172 | 0.172 | 0.172 | 0.172 | 0.172 | 0.172 | 0.172 | 0.172 | 0.186 | 0.174 | 0.169 | 0.201 | 0.151 | 0.159 | 0.165 | 0.165 | 0.165 | 0.165 | 0.852 | 0.852 |
| Cameroon |  |  |  |  |  | 0.015 | 0.015 | 0.021 | 0.021 | 0.016 | 0.029 | 0.028 | 0.028 | 0.028 | 0.028 | 0.028 | 0.037 | 0.037 | 0.037 | 0.116 |
| Canada |  |  |  |  |  |  |  |  |  |  |  |  |  |  |  |  | 6.393 | 6.413 | 6.4 | 6.4 |
| Central African Republic |  |  |  |  | 0.015 | 0.015 | 0.015 | 0.015 | 0.012 | 0.009 | 0.009 | 0.009 | 0.009 | 0.009 | 0.009 | 0.009 | 0.009 | 0.009 | 0.024 | 0.024 |
| Chad |  |  |  |  | 0.007 | 0.007 | 0.007 | 0.007 | 0.007 | 0.007 | 0.007 | 0.007 | 0.007 | 0.007 | 0.007 | 0.007 | 0.007 | 0.007 | 0.007 | 0.018 |
| Chile |  |  | 0.006 | 0.008 | 0.007 | 0.007 | 0.009 | 0.008 | 0.008 | 0.009 | 5.608 | 5.94 | 7.067 | 8.188 | 9.077 | 10.043 | 10.5 | 1.3 | 12.549 | 12.549 |
| China |  |  |  |  |  | 0.383 | 0.383 | 0.383 | 0.383 | 0.383 | 0.383 | 0.86 | 0.86 | 0.86 | 0.86 | 0.86 | 0.86 | 4.458 | 4.458 | 4.458 |
| Colombia | 7.657 | 7.878 | 8.306 | 8.317 | 8.539 | 8.763 | 8.989 | 9.217 | 9.447 | 9.682 | 9.919 | 9.919 | 9.919 | 9.919 | 9.919 | 9.743 | 9.743 | 10.1 | 10.1 | 10.1 |
| Comoros |  |  |  |  | 0.285 | 0.278 | 0.278 | 0.278 | 0.278 | 0.312 | 0.312 | 0.312 | 0.249 | 0.249 | 0.249 | 0.249 | 0.249 | 0.249 | 0.396 | 0.396 |
| Congo |  |  |  |  |  |  |  |  |  |  |  | 0.303 | 0.303 | 0.303 | 0.303 | 0.303 | 0.054 | 0.054 | 0.095 | 0.095 |
| Cook Islands |  | 5.55 | 5.55 | 5.55 | 5.293 | 5.293 | 5.293 | 5.293 | 5.293 | 2.15 | 2.15 | 2.15 | 2.15 | 2.15 | 12.44 | 12.44 | 12.44 | 12.44 | 12.44 | 3.419 |
| Costa Rica | 3.702 | 3.702 | 3.702 | 3.702 | 3.702 | 3.702 | 3.702 | 3.702 | 3.702 | 3.702 | 3.702 | 3.702 | 3.702 | 1.263 | 1.263 | 1.45 | 1.4 | 0.099 | 9.699 | 9.834 |
| Côte d'Ivoire |  |  |  |  | 0.189 | 0.111 | 0.111 | 0.111 | 0.14 | 0.14 | 0.14 | 0.14 | 0.14 | 0.14 | 0.216 | 0.216 | 0.216 | 0.216 | 0.137 | 0.145 |
| Croatia | 6.716 | 6.853 | 6.886 | 7.029 | 7.284 | 7.35 | 7.39 | 7.484 | 7.559 | 7.585 | 7.296 | 7.331 | 7.503 | 7.541 | 7.818 | 7.907 | 7.907 | 7.907 | 7.907 | 12.234 |
| Cuba | 8.913 | 8.847 | 8.889 | 9.054 | 9.29 | 9.372 | 9.547 | 9.676 | 9.997 | 10.308 | 10.818 | 11.385 | 12.435 | 13.515 | 14.708 | 14.708 | 14.708 | 16.812 | 16.699 | 16.699 |
| Cyprus | 6.562 | 6.602 | 6.653 | 6.753 | 6.898 | 7.084 | 6.839 | 6.797 | 6.87 | 6.894 | 6.939 | 6.961 | 7.189 | 7.247 | 7.281 | 7.545 | 7.545 | 7.545 | 8.039 | 8.039 |
| Czechia | 6.471 | 6.521 | 6.532 | 6.58 | 6.683 | 6.732 | 6.732 | 6.708 | 6.69 | 6.762 | 6.893 | 7.031 | 7.051 | 7.015 | 7.465 | 7.981 | 7.491 | 7.444 | 7.349 | 7.349 |
| Democratic People's Republic of Korea |  |  |  | 1.68 | 1.68 | 1.68 | 1.68 | 1.68 | 1.775 | 1.775 | 1.775 | 1.775 | 1.861 | 1.861 | 1.861 | 1.861 | 1.861 | 2.2 | 2.2 | 2.2 |
| Democratic Republic of the Congo |  |  |  |  |  |  |  |  |  | 0.007 | 0.007 | 0.007 | 0.007 | 0.007 | 0.007 | 0.007 | 0.019 | 0.019 | 0.019 | 0.082 |
| Denmark | 8.579 | 8.6 | 8.523 | 8.53 | 8.572 | 8.564 | 8.499 | 8.388 | 8.209 | 8.061 | 8.18 | 8.003 | 7.938 | 7.792 | 7.666 | 7.596 | 7.436 | 7.436 | 7.436 | 7.436 |
| Djibouti | 0 | 0 | 0 | 0 | 0.13 | 0.13 | 0.13 | 0.13 | 0.13 | 0.13 | 0.13 | 0.13 | 0.13 | 0.13 | 0.211 | 0.211 | 0.211 | 0.211 | 0.211 | 0.211 |
| Dominica | 0 | 0 | 0 | 0 | 0 | 0 | 0 | 0 | 0 | 0 | 0 | 0 | 0 | 0 | 0 | 0 | 0 | 0.7 | 1.117 | 1.117 |
| Dominican Republic | 8.263 | 8.263 | 8.263 | 8.263 | 8.263 | 8.263 | 8.263 | 8.263 | 1.274 | 1.864 | 1.864 | 1.946 | 1.946 | 1.946 | 1.946 | 1.946 | 1.946 | 2.151 | 2.199 | 1.888 |
| Ecuador | 1.626 | 1.64 | 1.697 | 1.655 | 1.655 | 1.655 | 1.655 | 1.655 | 1.655 | 2.276 | 2.276 | 2.744 | 2.744 | 2.744 | 2.744 | 5.282 | 3.179 | 3 | 3 | 3 |
| Egypt | 0 | 0 | 0 | 0 | 1.105 | 1.105 | 1.105 | 1.105 | 1.105 | 1.105 | 1.105 | 1.105 | 1.105 | 1.105 | 1.632 | 1.826 | 1.858 | 1.982 | 1.967 | 1.967 |
| El Salvador | 0 | 0 | 5.812 | 5.812 | 5.812 | 7.031 | 7.031 | 7.031 | 7.614 | 7.614 | 7.614 | 7.614 | 7.614 | 7.614 | 7.614 | 7.614 | 1.429 | 1.429 | 8.7 | 8.7 |
| Equatorial Guinea |  |  |  |  | 0.07 | 0.07 | 0.07 | 0.07 | 0.07 | 0.07 | 0.07 | 0.07 | 0.07 | 0.07 | 0.07 | 0.07 | 0.07 | 0.07 | 0.07 | 0.07 |
| Eritrea |  |  |  |  | 0.059 | 0.059 | 0.059 | 0.059 | 0.059 | 0.059 | 0.059 | 0.059 | 0.059 | 0.059 | 0.059 | 0.059 | 0.059 | 0.059 | 0.059 | 0.059 |
| Estonia | 7.526 | 8.055 | 7.803 | 8.209 | 8.184 | 8.778 | 8.849 | 8.659 | 9.231 | 8.951 | 8.993 | 8.88 | 9.039 | 9.022 | 9.291 | 9.42 | 9.548 | 9.588 | 9.653 | 9.653 |
| Eswatini | 0.03 | 0.03 | 0.03 | 0.03 | 0.166 | 0.166 | 0.166 | 0.166 | 0.166 | 0.123 | 0.123 | 0.429 | 0.429 | 0.429 | 0.429 | 0.429 | 0.429 | 0.429 | 0.429 | 0.122 |
| Ethiopia |  |  |  | 0.008 | 0.008 | 0.008 | 0.008 | 0.008 | 0.008 | 0.008 | 0.008 | 0.008 | 0.008 | 0.008 | 0.008 | 0.008 | 0.008 | 0.008 | 0.16 | 0.16 |
| Fiji |  |  |  | 0.735 | 0.735 | 0.735 | 0.735 | 0.735 | 0.735 | 2.003 | 2.003 | 2.003 | 2.003 | 2.003 | 2.003 | 0.737 | 0.737 | 0.737 | 0.737 | 1.202 |
| Finland | 8.5 | 8.768 | 8.935 | 8.935 | 8.126 | 8.053 | 7.854 | 7.805 | 7.533 | 7.431 | 7.891 | 7.775 | 7.404 | 7.259 | 7.302 | 7.302 | 7.302 | 7.302 | 8.148 | 8.148 |
| France | 7.214 | 7.183 | 7.165 | 7.153 | 7.132 | 7.129 | 7.093 | 7.047 | 6.962 | 6.894 | 6.862 | 6.565 | 6.567 | 6.587 | 6.586 | 6.61 | 6.653 | 6.657 | 6.668 | 6.668 |
| Gabon |  |  |  |  | 0.103 | 0.103 | 0.103 | 0.103 | 0.198 | 0.198 | 0.198 | 0.198 | 0.198 | 0.198 | 0.198 | 0.198 | 0.045 | 0.179 | 0.179 | 0.212 |
| Gambia |  |  |  | 0.048 | 0.048 | 0.065 | 0.063 | 0.067 | 0.071 | 0.071 | 0.071 | 0.071 | 0.071 | 0.071 | 0.071 | 0.082 | 0.082 | 0.082 | 0.082 | 0.082 |
| Georgia | 3.448 | 3.107 | 3.297 | 3.322 | 3.01 | 3.245 | 2.964 | 2.877 | 2.817 | 2.631 | 3.067 | 4.271 | 4.943 | 4.979 | 5.333 | 6.135 | 6.39 | 6.162 | 7.619 | 7.704 |
| Germany | 7.551 | 7.611 | 7.635 | 7.716 | 7.768 | 7.796 | 7.838 | 7.919 | 8.011 | 8.124 | 8.218 | 8.283 | 8.375 | 8.417 | 8.498 | 8.555 | 8.577 | 8.564 | 8.553 | 8.553 |
| Ghana |  |  |  |  | 0.049 | 0.049 | 0.049 | 0.013 | 0.019 | 0.019 | 0.019 | 0.019 | 0.019 | 0.019 | 0.019 | 0.019 | 0.14 | 0.14 | 0.14 | 0.14 |
| Greece |  |  |  |  |  |  |  |  |  |  | 13.466 | 13.407 | 13.179 | 12.952 | 12.845 | 12.478 | 12.459 | 12.547 | 12.547 | 12.547 |
| Grenada | 0.875 | 0.872 | 0.868 | 0.865 | 0.865 | 0.865 | 1.811 | 1.811 | 1.811 | 1.811 | 1.811 | 1.811 | 1.811 | 1.811 | 1.811 | 1.811 | 1.811 | 1.533 | 1.974 | 1.974 |
| Guatemala |  |  |  |  |  |  |  | 1.734 | 1.734 | 1.734 | 1.734 | 1.734 | 1.734 | 1.734 | 1.734 | 1.734 | 1.734 | 0.1 | 0.104 | 0.104 |
| Guinea | 0.022 | 0.022 | 0.022 | 0.022 | 0.025 | 0.019 | 0.019 | 0.019 | 0.019 | 0.019 | 0.019 | 0.019 | 0.019 | 0.019 | 0.019 | 0.019 | 0.043 | 0.043 | 0.043 | 0.043 |
| Guinea-Bissau |  |  |  |  | 0.023 | 0.023 | 0.023 | 0.023 | 0.014 | 0.047 | 0.047 | 0.047 | 0.047 | 0.047 | 0.047 | 0.047 | 0.047 | 0.047 | 0.037 | 0.037 |
| Guyana | 0.402 | 0.402 | 0.402 | 0.402 | 0.402 | 0.402 | 0.402 | 0.402 | 0.402 | 0.388 | 0.388 | 0.388 | 0.388 | 0.388 | 0.388 | 0.388 | 0.388 | 0.388 | 1.194 | 1.194 |
| Haiti | 0 | 0 | 0 | 0 | 0 | 0 | 0 | 0 | 0 | 0 | 0 | 0.05 | 0.05 | 0.1 | 0.1 | 0.058 | 0.058 | 0.058 | 0.213 | 0.213 |
| Honduras | 1.735 | 1.735 | 1.735 | 1.735 | 1.735 | 1.735 | 1.735 | 1.735 | 0.296 | 0.296 | 0.296 | 0.296 | 0.296 | 0.296 | 0.296 | 0.296 | 2.628 | 0.327 | 0.327 | 0.327 |
| Hungary | 3.234 | 3.571 | 4.763 | 5.277 | 5.092 | 4.47 | 4.969 | 4.003 | 5.068 | 4.94 | 5.295 | 5.291 | 5.686 | 6.064 | 6.326 | 6.071 | 6.237 | 6.772 | 7.077 | 7.077 |
| Iceland | 10.09 | 10.07 | 10.09 | 10.00 | 9.859 | 9.797 | 9.542 | 9.634 | 9.683 | 9.302 | 9.334 | 8.749 | 8.291 | 8.313 | 8.339 | 8.418 | 8.368 | 8.343 | 8.583 | 8.672 |
| India | 0.37 | 0.439 | 0.431 | 0.426 | 0.487 | 0.482 | 0.67 | 0.617 | 0.777 | 0.859 | 0.859 | 0.942 | 0.955 | 0.955 | 1.192 | 1.192 | 1.493 | 1.877 | 1.611 | 1.989 |
| Indonesia |  |  |  | 0.239 | 0.239 | 0.239 | 0.239 | 0.239 | 0.239 | 0.228 | 0.361 | 0.361 | 0.425 | 0.425 | 0.425 | 0.493 | 0.493 | 0.546 | 0.574 | 0.582 |
| Iran (Islamic Republic of) |  |  |  |  | 1.544 | 1.894 | 1.35 | 1.35 | 1.35 | 1.35 | 1.35 | 1.35 | 1.35 | 1.35 | 3.586 | 3.336 | 3.336 | 3.268 | 4.474 | 4.474 |
| Iraq |  |  |  |  |  |  |  |  |  |  | 1.614 | 1.614 | 1.614 | 1.614 | 2.293 | 2.293 | 2.439 | 2.536 | 2.536 | 2.639 |
| Ireland | 5.02 | 5.221 | 5.379 | 5.455 | 5.512 | 5.619 | 5.706 | 5.866 | 6.146 | 6.012 | 5.975 | 5.809 | 5.774 | 5.739 | 5.961 | 6.079 | 6.28 | 6.549 | 6.676 | 6.676 |
| Israel | 8.409 | 8.469 | 9.186 | 9.823 | 9.275 | 8.627 | 8.333 | 8.325 | 8.357 | 8.484 | 8.667 | 7.836 | 7.048 | 6.981 | 8.021 | 8.314 | 8.056 | 7.278 | 7.278 | 7.278 |
| Italy |  |  |  |  |  |  |  |  |  |  |  |  |  | 7.818 | 7.881 | 7.858 | 8.005 | 8.167 | 8.236 | 8.236 |
| Jamaica |  |  |  | 0.782 | 0.782 | 0.782 | 0.782 | 0.782 | 0.165 | 0.165 | 0.165 | 0.165 | 0.123 | 0.122 | 0.125 | 0.128 | 0.155 | 0.897 | 0.897 | 0.897 |
| Japan | 7.125 | 7.125 | 7.262 | 7.262 | 7.425 | 7.425 | 7.367 | 7.367 | 7.522 | 7.522 | 7.681 | 7.681 | 7.762 | 7.762 | 7.88 | 7.88 | 7.951 | 7.951 | 7.951 | 7.951 |
| Jordan | 5.632 | 5.499 | 4.48 | 6.389 | 7.12 | 7.274 | 7.672 | 7.819 | 7.769 | 6.358 | 7.837 | 7.837 | 7.837 | 7.987 | 7.715 | 7.345 | 7.026 | 7.263 | 7.132 | 7.132 |
| Kazakhstan | 2.649 | 2.909 | 3.343 | 3.454 | 3.555 | 3.392 | 3.609 | 3.641 | 3.553 | 3.547 | 4.096 | 3.961 | 4.131 | 3.762 | 3.716 | 2.896 | 2.896 | 2.896 | 2.896 | 2.896 |
| Kenya |  |  | 0.221 | 0.221 | 0.221 | 0.221 | 0.221 | 0.221 | 0.245 | 0.245 | 0.214 | 0.215 | 0.222 | 0.23 | 0.233 | 0.233 | 0.233 | 0.233 | 0.164 | 0.247 |
| Kiribati |  |  |  |  | 0.331 | 0.331 | 0.331 | 0.331 | 1.823 | 1.823 | 0.389 | 0.286 | 0.286 | 0.649 | 0.649 | 0.649 | 0.711 | 0.711 | 0.711 | 0.711 |
| Kuwait |  | 3.2 | 3.2 | 3.2 | 3.2 | 3.2 | 3.126 | 3.395 | 3.618 | 3.736 | 5.461 | 5.795 | 6.122 | 6.233 | 6.147 | 6.745 | 6.745 | 6.745 | 6.745 | 6.745 |
| Kyrgyzstan | 2.276 | 2.17 | 2.11 | 1.979 | 2.104 | 2.057 | 1.985 | 1.969 | 1.905 | 1.944 | 1.885 | 1.731 | 1.731 | 1.712 | 1.651 | 1.651 | 1.651 | 1.651 | 1.85 | 1.85 |
| Lao People's Democratic Republic |  |  |  |  |  |  |  |  |  | 0.371 | 0.371 | 0.371 | 0.498 | 0.498 | 0.514 | 0.514 | 0.514 | 0.71 | 0.622 | 0.622 |
| Latvia | 5.188 | 5.257 | 5.406 | 5.521 | 5.998 | 6.31 | 6.476 | 6.656 | 6.978 | 7.04 | 7.023 | 6.955 | 6.984 | 7.135 | 6.927 | 7.103 | 7.147 | 7.073 | 7.057 | 7.138 |
| Lebanon |  | 10.732 | 10.732 | 10.732 | 10.732 | 10.732 | 8.526 | 8.81 | 8.81 | 10.314 | 10.892 | 12.036 | 12.036 | 8.913 | 8.735 | 8.461 | 9.822 | 9.865 | 10.206 | 10.206 |
| Lesotho |  |  |  | 0.015 | 0.015 | 0.015 | 0.015 | 0.015 | 0.015 | 0.015 | 0.015 | 0.015 | 0.015 | 0.015 | 0.015 | 0.015 | 0.015 | 0.015 | 0.015 | 0.174 |
| Liberia |  |  |  |  | 0.01 | 0.01 | 0.01 | 0.01 | 0.011 | 0.061 | 0.059 | 0.059 | 0.059 | 0.059 | 0.059 | 0.059 | 0.059 | 0.059 | 0.059 | 0.014 |
| Libya |  |  |  |  | 1.489 | 1.489 | 1.489 | 1.489 | 3.377 | 6.182 | 6.182 | 6.182 | 6.182 | 6.182 | 7.204 | 7.77 | 7.77 | 8.777 | 8.777 | 8.777 |
| Lithuania | 6.842 | 6.952 | 6.494 | 6.719 | 6.477 | 7.081 | 6.533 | 7.122 | 6.866 | 7.41 | 7.862 | 8.063 | 8.816 | 8.9 | 8.982 | 9.018 | 9.645 | 9.977 | 9.977 | 9.977 |
| Luxembourg | 5.962 | 6.247 | 6.49 | 6.55 | 7.835 | 8.081 | 8.054 | 8.128 | 8.199 | 8.217 | 8.309 | 8.396 | 8.307 | 8.478 | 8.584 | 8.928 | 9.495 | 9.816 | 9.816 | 6.172 |
| Madagascar |  |  | 0.071 | 0.092 | 0.09 | 0.107 | 0.104 | 0.1 | 0.105 | 0.087 | 0.092 | 0.083 | 0.081 | 0.048 | 0.048 | 0.048 | 0.048 | 0.048 | 0.473 | 0.473 |
| Malawi |  |  |  |  |  |  |  |  | 0.127 | 0.127 | 0.127 | 0.127 | 0.127 | 0.127 | 0.127 | 0.127 | 0.127 | 0.127 | 0.017 | 0.017 |
| Malaysia | 0.924 | 0.924 | 0.892 | 0.892 | 0.892 | 0.892 | 0.892 | 0.892 | 1.336 | 1.336 | 1.351 | 1.484 | 1.568 | 1.568 | 1.568 | 3.5 | 3.5 | 3.5 | 3.082 | 3.082 |
| Maldives |  |  |  |  | 0.321 | 0.321 | 0.321 | 0.321 | 0.321 | 1.273 | 0.875 | 0.875 | 0.875 | 0.875 | 0.875 | 1.363 | 0.21 | 1.672 | 1.997 | 1.997 |
| Mali |  |  |  |  | 0.036 | 0.036 | 0.036 | 0.036 | 0.036 | 0.027 | 0.023 | 0.023 | 0.023 | 0.023 | 0.023 | 0.023 | 0.06 | 0.06 | 0.053 | 0.053 |
| Malta |  |  |  |  |  |  |  |  |  | 4.349 | 4.442 | 4.453 | 4.504 | 4.623 | 4.672 | 4.751 | 4.751 | 4.751 | 4.751 | 4.837 |
| Marshall Islands |  |  |  |  |  |  |  | 1.071 | 1.071 | 1.071 | 0.71 | 0.71 | 0.705 | 0.705 | 0.705 | 0.705 | 0.705 | 0.705 | 0.705 | 1.191 |
| Mauritania |  |  |  |  | 0.16 | 0.16 | 0.16 | 0.16 | 0.16 | 0.206 | 0.206 | 0.206 | 0.206 | 0.206 | 0.206 | 0.206 | 0.206 | 0.206 | 0.211 | 0.53 |
| Mauritius |  |  |  |  | 1.374 | 1.374 | 1.374 | 1.848 | 1.896 | 2.01 | 2.083 | 2.142 | 2.401 | 2.796 | 2.911 | 3.017 | 3.051 | 3.171 | 3.171 | 2.764 |
| Mexico | 6.035 | 6.035 | 6.035 | 6.035 | 6.035 | 0.939 | 0.97 | 1.003 | 1.048 | 1.076 | 1.159 | 1.163 | 1.166 | 1.22 | 1.279 | 1.304 | 1.362 | 1.368 | 1.368 | 1.368 |
| Micronesia (Federated States of) | 1.303 | 1.303 | 1.303 | 0.935 | 0.935 | 1.225 | 1.225 | 1.225 | 1.352 | 1.352 | 1.352 | 1.352 | 1.352 | 1.352 | 1.352 | 1.352 | 1.352 | 1.352 | 1.352 | 1.352 |
| Monaco |  |  |  |  |  |  |  |  |  |  |  | 10.548 | 10.148 | 10.298 | 10.182 | 10.182 | 10.182 | 10.182 | 10.182 | 10.182 |
| Mongolia |  |  | 1.289 | 1.289 | 1.289 | 1.289 | 1.289 | 1.289 | 1.949 | 1.975 | 1.96 | 2.353 | 2.818 | 2.818 | 2.818 | 2.818 | 2.339 | 2.339 | 4.082 | 4.082 |
| Montenegro |  |  |  |  |  |  |  |  |  |  | 0.416 | 0.384 | 0.399 | 0.399 | 0.399 | 0.399 | 0.399 | 0.399 | 0.462 | 0.462 |
| Morocco |  |  |  |  | 1.026 | 1.026 | 1.026 | 1.026 | 1.026 | 0.836 | 0.836 | 0.836 | 0.836 | 0.836 | 1.361 | 1.406 | 1.406 | 1.364 | 1.364 | 1.364 |
| Mozambique |  |  |  |  | 0.008 | 0.008 | 0.008 | 0.008 | 0.008 | 0.023 | 0.025 | 0.031 | 0.028 | 0.053 | 0.053 | 0.07 | 0.07 | 0.072 | 0.086 | 0.095 |
| Myanmar |  |  |  |  | 0.274 | 0.326 | 0.351 | 0.376 | 0.462 | 0.459 | 0.506 | 0.543 | 0.586 | 0.586 | 0.586 | 0.621 | 0.662 | 0.806 | 0.652 | 0.892 |
| Namibia |  |  |  |  | 0.43 | 0.43 | 0.43 | 0.449 | 0.449 | 0.449 | 0.449 | 0.449 | 0.449 | 0.449 | 0.299 | 0.299 | 0.299 | 0.299 | 0.727 | 0.727 |
| Nauru |  |  |  |  | 1.009 | 1.009 | 1.009 | 1.009 | 1.012 | 1.006 | 1.006 | 0.993 | 0.993 | 0.993 | 0.993 | 1.928 | 1.928 | 1.928 | 1.928 | 3.716 |
| Nepal |  |  |  |  | 0.096 | 0.096 | 0.096 | 0.096 | 0.096 | 0.096 | 0.096 | 0.096 | 0.096 | 0.096 | 0.096 | 0.096 | 0.096 | 2.2 | 0.967 | 0.967 |
| Netherlands |  |  |  |  |  | 4.097 | 4.285 | 4.444 | 4.463 | 4.601 | 4.699 | 4.914 | 4.937 | 4.937 | 4.979 | 5.154 | 5.126 | 5.073 | 5.073 | 6.548 |
| New Zealand |  | 3.662 | 4.088 | 4.088 | 4.088 | 3.961 | 4.083 | 4.434 | 4.476 | 4.471 | 5.849 | 5.927 | 5.96 | 6.066 | 6.102 | 6.12 | 6.179 | 6.244 | 5.053 | 5.053 |
| Nicaragua |  |  |  | 0.459 | 0.459 | 0.452 | 0.443 | 0.436 | 0.469 | 0.461 | 0.443 | 0.446 | 0.441 | 0.434 | 0.423 | 0.423 | 0.423 | 0.423 | 0.399 | 0.399 |
| Niger |  |  |  |  | 0.011 | 0.011 | 0.011 | 0.011 | 0.01 | 0.011 | 0.011 | 0.011 | 0.011 | 0.016 | 0.011 | 0.015 | 0.009 | 0.009 | 0.009 | 0.009 |
| Nigeria |  |  |  | 0.188 | 0.188 | 0.152 | 0.157 | 0.176 | 0.171 | 0.16 | 0.527 | 0.527 | 0.527 | 0.527 | 0.527 | 0.527 | 0.219 | 0.219 | 0.222 | 0.222 |
| Niue |  |  |  | 11.44 | 11.68 | 11.68 | 18.00 | 18.00 | 12.25 | 12.25 | 12.25 | 12.25 | 12.25 | 12.25 | 12.25 | 12.25 | 12.25 | 12.25 | 12.25 | 12.25 |
| North Macedonia |  |  |  |  |  |  |  |  |  | 6.887 | 7.722 | 7.826 | 7.964 | 8.213 | 8.213 | 8.772 | 8.772 | 8.772 | 8.772 | 8.772 |
| Norway | 8.019 | 8.021 | 8.066 | 8.109 | 8.023 | 8.406 | 8.782 | 8.692 | 8.778 | 8.685 | 8.787 | 8.851 | 8.656 | 8.729 | 8.654 | 8.527 | 8.621 | 8.666 | 8.696 | 8.743 |
| Oman | 1.155 | 1.155 | 1.155 | 1.155 | 1.701 | 1.784 | 1.922 | 1.972 | 2.025 | 2.166 | 2.15 | 2.233 | 2.301 | 2.329 | 2.558 | 2.693 | 2.755 | 2.893 | 2.975 | 3.003 |
| Pakistan | 0.293 | 0.317 | 0.317 | 0.317 | 0.412 | 0.412 | 0.412 | 0.412 | 0.525 | 0.56 | 0.586 | 0.635 | 0.678 | 0.717 | 0.773 | 0.835 | 0.9 | 0.984 | 0.964 | 1.232 |
| Palau |  |  |  |  |  |  |  | 2.61 | 2.61 | 2.61 | 2.61 | 2.61 | 2.61 | 2.61 | 2.61 | 2.61 | 2.61 | 2.61 | 2.61 | 2.222 |
| Panama |  | 2.476 | 2.848 | 2.705 | 2.823 | 2.817 | 2.81 | 2.733 | 2.816 | 2.883 | 2.995 | 3.006 | 3.267 | 2.704 | 2.704 | 2.704 | 2.797 | 3 | 3 | 3 |
| Papua New Guinea | 0.154 | 0.154 | 0.154 | 0.154 | 0.154 | 0.154 | 0.154 | 0.154 | 0.154 | 0.169 | 0.169 | 0.169 | 0.169 | 0.169 | 0.169 | 0.169 | 0.169 | 0.169 | 0.165 | 0.071 |
| Paraguay |  |  | 4.385 | 4.385 | 4.385 | 4.385 | 4.385 | 4.385 | 4.385 | 4.385 | 4.385 | 4.385 | 1.641 | 1.641 | 1.641 | 1.641 | 1.641 | 1.641 | 1.623 | 1.623 |
| Peru |  |  |  |  |  |  |  | 5.407 | 5.407 | 1.24 | 1.24 | 1.24 | 1.515 | 1.515 | 1.515 | 1.515 | 1.849 | 1.849 | 1.5 | 1.5 |
| Philippines | 1.098 | 1.098 | 5.312 | 5.312 | 5.419 | 5.419 | 5.419 | 5.419 | 0.208 | 0.208 | 0.208 | 0.208 | 0.208 | 0.208 | 0.208 | 0.021 | 0.019 | 2.429 | 2.429 | 2.604 |
| Poland | 3.05 | 2.628 | 2.8 | 2.886 | 3.682 | 3.192 | 3.276 | 3.481 | 3.399 | 3.173 | 3.274 | 3.404 | 3.268 | 3.229 | 3.436 | 3.314 | 3.503 | 3.512 | 3.512 | 9.828 |
| Portugal |  |  | 3.98 | 4.223 | 4.495 | 4.811 | 5.373 | 5.324 | 5.694 | 6.229 | 7.649 | 7.671 | 8.042 | 8.467 | 8.759 | 9.132 | 9.564 | 10.12 | 9.586 | 9.586 |
| Qatar |  |  |  |  |  | 7.973 | 4.752 | 4.752 | 4.752 | 4.752 | 4.752 | 4.752 | 4.752 | 4.752 | 6.758 | 6.758 | 8.119 | 8.119 | 6.259 | 6.147 |
| Republic of Korea |  |  |  |  |  | 3.649 | 3.788 | 3.894 | 4.016 | 4.137 | 4.226 | 4.3 | 4.372 | 4.466 | 4.535 | 4.632 | 4.737 | 4.951 | 6.04 | 6.04 |
| Republic of Moldova | 3.165 | 3.107 | 3.262 | 3.29 | 3.364 | 3.493 | 3.56 | 3.717 | 3.859 | 3.888 | 4.016 | 4.099 | 4.097 | 4.297 | 4.299 | 4.368 | 4.368 | 4.215 | 4.215 | 1.801 |
| Romania | 3.598 | 3.733 | 3.821 | 4.153 | 4.394 | 4.773 | 4.986 | 5.52 | 5.691 | 6.032 | 6.33 | 6.552 | 6.812 | 7.077 | 7.077 | 7.077 | 8.065 | 7.964 | 7.964 | 7.964 |
| Russian Federation | 2.972 | 2.961 | 2.975 | 2.949 | 2.945 | 2.901 | 2.913 | 2.888 | 2.895 | 2.865 | 2.891 | 2.921 | 2.933 | 2.892 | 2.842 | 2.842 | 2.842 | 2.842 | 2.842 | 2.842 |
| Rwanda |  |  |  |  |  |  |  |  | 0.066 | 0.074 | 0.074 | 0.074 | 0.074 | 0.074 | 0.074 | 0.112 | 0.112 | 0.197 | 0.185 | 0.185 |
| Saint Kitts and Nevis |  | 3.363 | 3.363 | 3.363 | 3.363 | 3.363 | 3.363 | 3.363 | 3.363 | 3.363 | 3.363 | 3.363 | 3.363 | 3.363 | 3.363 | 3.906 | 3.906 | 3.906 | 2.288 | 2.288 |
| Saint Lucia |  | 1.707 | 0.439 | 0.436 | 0.433 | 0.433 | 0.433 | 0.179 | 0.118 | 0.29 | 1.608 | 2.393 | 2.774 | 3.042 | 2.187 | 2.187 | 2.187 | 1.713 | 1.713 | 1.713 |
| Saint Vincent and the Grenadines | 0.464 | 1.946 | 1.946 | 1.946 | 1.198 | 1.198 | 1.198 | 1.198 | 1.198 | 1.198 | 1.198 | 1.198 | 1.66 | 1.66 | 1.66 | 1.66 | 1.66 | 1.66 | 1.66 | 1.66 |
| Samoa |  |  |  | 0.563 | 0.563 | 0.563 | 0.563 | 0.563 | 0.928 | 0.928 | 2.42 | 2.42 | 2.42 | 0.629 | 0.629 | 0.629 | 1.491 | 1.491 | 1.491 | 1.065 |
| San Marino |  |  |  |  |  |  |  |  |  |  |  |  |  |  | 6.679 | 6.679 | 6.679 | 6.679 | 17.76 | 17.76 |
| Sao Tome and Principe |  |  |  |  |  |  |  |  |  |  |  |  |  |  |  |  |  |  |  | 0.186 |
| Saudi Arabia |  | 0.746 | 0.746 | 0.746 | 0.746 | 0.746 | 0.746 | 0.746 | 0.746 | 0.746 | 0.746 | 0.746 | 0.746 | 0.746 | 3.979 | 3.979 | 4.377 | 4.21 | 4.971 | 4.971 |
| Senegal |  |  |  |  | 0.065 | 0.065 | 0.065 | 0.065 | 0.087 | 0.087 | 0.087 | 0.087 | 0.087 | 0.039 | 0.039 | 0.039 | 0.081 | 0.078 | 0.078 | 0.354 |
| Serbia |  |  |  |  |  |  |  |  |  | 2.519 | 2.814 | 2.845 | 2.749 | 2.685 | 2.596 | 2.142 | 2.142 | 2.142 | 2.142 | 2.142 |
| Seychelles |  |  |  |  | 1.488 | 1.128 | 1.675 | 1.776 | 1.327 | 1.762 | 2.082 | 1.959 | 1.512 | 1.512 | 1.512 | 1.512 | 3.552 | 3.552 | 3.552 | 4.297 |
| Sierra Leone |  |  |  |  | 0.009 | 0.009 | 0.009 | 0.009 | 0.01 | 0.01 | 0.009 | 0.009 | 0.009 | 0.009 | 0.009 | 0.009 | 0.072 | 0.072 | 0.072 | 0.072 |
| Singapore |  | 2.666 | 2.666 | 2.882 | 2.937 | 2.994 | 3.005 | 2.957 | 2.961 | 2.946 | 3.056 | 2.808 | 2.928 | 3.308 | 3.308 | 3.308 | 3.888 | 3.888 | 4.104 | 4.104 |
| Slovakia | 4.415 | 4.391 | 4.333 | 4.33 | 4.33 | 4.33 | 4.33 | 4.33 | 4.33 | 4.875 | 4.875 | 4.875 | 4.875 | 4.77 | 4.77 | 4.87 | 4.87 | 4.87 | 4.87 | 6.916 |
| Slovenia | 5.831 | 5.927 | 6.033 | 6.052 | 5.994 | 6.005 | 6.003 | 6.133 | 6.011 | 6.077 | 6.161 | 6.24 | 6.298 | 6.48 | 6.602 | 6.721 | 6.851 | 7.007 | 7.007 | 7.007 |
| Solomon Islands |  |  |  |  |  | 1.107 | 1.107 | 1.107 | 1.107 | 1.107 | 1.107 | 0.665 | 0.396 | 0.473 | 0.473 | 0.473 | 0.468 | 0.468 | 0.468 | 0.746 |
| Somalia |  |  |  |  |  |  |  |  |  |  |  |  |  |  |  |  |  |  |  |  |
| South Africa |  |  |  |  | 1.179 | 1.179 | 1.179 | 1.179 | 1.179 | 1.179 | 1.034 | 1.036 | 1.051 | 1.075 | 1.066 | 1.081 | 1.096 | 1.108 | 1.113 | 1.087 |
| South Sudan |  |  |  |  |  |  |  |  |  |  |  |  |  |  |  |  |  |  | 0.003 | 0.003 |
| Spain | 4.296 | 4.479 | 4.602 | 4.696 | 4.862 | 5.032 | 5.209 | 5.396 | 5.578 | 5.737 | 5.929 | 6.174 | 6.627 | 6.913 | 7.116 | 7.422 | 7.659 | 7.865 | 0 | 8.216 |
| Sri Lanka | 0.339 | 0.339 | 0.394 | 0.394 | 0.398 | 0.488 | 0.6 | 0.662 | 0.658 | 0.426 | 0.516 | 0.562 | 0.596 | 0.619 | 0.654 | 0.65 | 0.686 | 0.697 | 0.65 | 0.961 |
| Sudan | 0 | 0 | 0 | 0 | 0.232 | 0.232 | 0.298 | 0.233 | 0.234 | 0.234 | 0.234 | 0.234 | 0.234 | 0.234 | 0.234 | 2.086 | 2.086 | 2.086 | 2.086 | 2.086 |
| Suriname | 0.085 | 0.085 | 0.085 | 0.085 | 0.851 | 0.851 | 0.851 | 0.851 | 0.851 | 0.478 | 0.478 | 0.478 | 0.478 | 0.478 | 0.478 | 0.478 | 0.478 | 0.478 | 0.694 | 0.694 |
| Sweden | 7.966 | 8.024 | 8.094 | 8.064 | 8.181 | 8.113 | 8.145 | 8.123 | 7.972 | 7.902 | 7.927 | 7.989 | 8.009 | 7.974 | 7.962 | 8.001 | 8.212 | 8.212 | 8.212 | 8.212 |
| Switzerland | 4.855 | 4.78 | 4.849 | 4.95 | 5.023 | 5.096 | 5.208 | 5.216 | 5.23 | 5.224 | 5.262 | 5.214 | 5.221 | 5.189 | 5.094 | 5.062 | 5.032 | 5.032 | 5.032 | 5.032 |
| Syrian Arab Republic | 6.8 | 6.8 | 8.552 | 8.565 | 8.589 | 8.564 | 8.564 | 7.441 | 7.825 | 7.45 | 7.482 | 7.482 | 7.482 | 7.482 | 8.864 | 8.864 | 7.175 | 7.175 | 7.175 | 7.175 |
| Tajikistan | 1.483 | 1.464 | 1.486 | 1.423 | 1.457 | 1.46 | 1.429 | 1.465 | 1.481 | 1.532 | 1.639 | 1.588 | 1.543 | 1.613 | 1.562 | 1.562 | 1.562 | 1.562 | 1.562 | 1.562 |
| Thailand |  | 0.679 | 0.698 | 0.698 | 0.635 | 0.635 | 1.338 | 1.411 | 1.45 | 1.484 | 1.763 | 1.763 | 1.763 | 1.763 | 1.763 | 1.763 | 1.618 | 1.672 | 2.319 | 2.377 |
| Timor-Leste |  | 0.011 | 0.011 | 0.011 | 0.021 | 0.021 | 0.021 | 0.021 | 0.019 | 0.056 | 0.064 | 0.063 | 0.063 | 0.063 | 0.153 | 0.15 | 0.15 | 0.684 | 0.063 | 0.077 |
| Togo |  |  |  |  | 0.033 | 0.033 | 0.033 | 0.025 | 0.025 | 0.025 | 0.103 | 0.103 | 0.103 | 0.103 | 0.103 | 0.025 | 0.025 | 0.074 | 0.027 | 0.027 |
| Tonga |  | 3.351 | 3.351 | 2.309 | 2.309 | 2.309 | 2.309 | 2.309 | 2.309 | 1.059 | 0.962 | 0.962 | 0.962 | 1.179 | 1.179 | 1.179 | 1.179 | 1.179 | 1.179 | 1.627 |
| Trinidad and Tobago | 1.563 | 1.714 | 1.723 | 1.94 | 1.94 | 1.94 | 2.18 | 2.247 | 2.247 | 2.362 | 2.583 | 2.567 | 2.567 | 2.567 | 2.567 | 3.568 | 3.568 | 3.569 | 3.396 | 3.183 |
| Tunisia | 1.355 | 1.409 | 1.412 | 1.412 | 2.095 | 1.83 | 1.821 | 1.821 | 1.821 | 1.821 | 2.943 | 2.943 | 2.943 | 2.943 | 2.943 | 3.125 | 3.059 | 3.059 | 3.059 | 3.059 |
| Turkey | 2.515 | 2.504 | 2.513 | 2.685 | 2.67 | 2.673 | 2.666 | 2.771 | 2.834 | 2.887 | 2.963 | 2.873 | 2.867 | 2.936 | 2.978 | 3.162 | 3.341 | 3.438 | 3.438 | 3.438 |
| Turkmenistan | 2.407 | 2.303 | 2.161 | 2.111 | 1.641 | 1.592 | 1.462 | 1.439 | 1.41 | 1.402 | 1.152 | 1.167 | 1.166 | 1.137 | 1.154 | 1.154 | 1.154 | 1.154 | 1.154 | 1.154 |
| Tuvalu |  |  | 2.085 | 2.057 | 2.057 | 2.057 | 2.057 | 2.057 | 1.939 | 1.939 | 1.939 | 1.939 | 1.939 | 1.939 | 4.557 | 4.557 | 4.557 | 4.557 | 4.557 | 4.557 |
| Uganda |  |  |  |  | 0.044 | 0.035 | 0.035 | 0.035 | 0.035 | 0.035 | 0.035 | 0.035 | 0.035 | 0.035 | 0.035 | 0.072 | 0.072 | 0.072 | 0.072 | 0.07 |
| Ukraine | 4.581 | 4.544 | 4.565 | 4.588 | 4.594 | 4.63 | 4.598 | 4.605 | 4.604 | 6.459 | 6.583 | 6.632 | 6.751 | 6.831 | 5.975 | 5.975 | 5.975 | 5.975 | 5.975 | 5.975 |
| United Arab Emirates | 2.559 | 3.164 | 3.306 | 3.432 | 3.647 | 3.082 | 2.562 | 3.111 | 2.996 | 3.63 | 3.604 | 3.353 | 3.438 | 3.458 | 4.351 | 5.307 | 5.518 | 5.914 | 6.513 | 6.513 |
| United Kingdom of Great Britain and Northern Ireland |  |  |  |  |  |  |  | 4.792 | 4.927 | 4.997 | 5.09 | 5.189 | 5.156 | 5.182 | 5.25 | 5.257 | 5.259 | 5.209 | 5.213 | 5.213 |
| United Republic of Tanzania |  |  | 0.042 | 0.042 | 0.042 | 0.042 | 0.011 | 0.011 | 0.011 | 0.011 | 0.011 | 0.011 | 0.021 | 0.021 | 0.021 | 0.021 | 0.021 | 0.021 | 0.075 | 0.075 |
| United States of America | 4.765 | 4.765 | 4.765 | 4.765 | 4.765 | 6.082 | 6.082 | 6.082 | 6.082 | 6.082 | 5.778 | 5.778 | 5.778 | 5.778 | 5.778 | 5.778 | 5.778 | 5.778 | 6.1 | 6.1 |
| Uruguay |  |  | 11.834 | 11.834 | 11.834 | 11.834 | 11.834 | 11.834 | 7.413 | 7.413 | 7.413 | 7.413 | 7.413 | 7.413 | 7.413 | 7.413 | 12.193 | 14.5 | 14.5 | 14.5 |
| Uzbekistan | 2.063 | 2.104 | 2.11 | 2.085 | 2.015 | 1.965 | 1.793 | 1.745 | 1.634 | 1.592 | 1.646 | 1.678 | 1.602 | 1.587 | 1.486 | 1.486 | 1.486 | 1.486 | 1.486 | 1.486 |
| Vanuatu |  |  |  |  |  |  |  |  | 0.134 | 0.134 | 0.134 | 0.134 | 0.681 | 0.681 | 0.681 | 0.681 | 0.681 | 0.681 | 0.681 | 0.3 |
| Venezuela (Bolivarian Republic of) |  | 5.55 | 5.55 | 5.55 | 5.55 | 5.55 | 5.55 | 5.55 | 5.55 | 5.55 | 5.55 | 5.55 | 5.55 | 5.55 | 5.55 | 5.55 | 5.55 | 1.4 | 1.4 | 1.4 |
| Yemen |  |  |  |  | 0.245 | 0.245 | 0.245 | 0.245 | 0.245 | 0.245 | 0.247 | 0.247 | 0.247 | 0.247 | 0.21 | 0.21 | 0.21 | 0.21 | 0.21 | 0.21 |
| Zambia |  |  |  |  | 0.233 | 0.233 | 0.233 | 0.233 | 0.233 | 0.182 | 0.181 | 0.052 | 0.053 | 0.053 | 0.053 | 0.053 | 0.191 | 0.191 | 0.084 | 0.084 |
| Zimbabwe |  |  |  |  |  |  |  | 0.157 | 0.146 | 0.145 | 0.035 | 0.198 | 0.178 | 0.177 | 0.079 | 0.048 | 0.097 | 0.077 | 0.072 | 0.169 |

Source: The workforce data are based on the latest available data in the NHWA data platform as of 31 March 2021, apart from the data for 2019, which a combination of the latest available data from the NHWA data platform and King’s College London survey was used.
