# Supplementary material for: Health workforce for oral health inequity: Opportunity for action
Source: PLoS One. 2024 Jun 13;19(6):e0292549. doi: 10.1371/journal.pone.0292549 (PMC11175420; doi:10.1371/journal.pone.0292549)
Supplement: S3 Table — Note: further information available on request from authors. (DOCX) [file pone.0292549.s005.docx]

**S5: Supplementary Information: dental Schools (public and private) by region and country income status**

**Table S5a. Distribution of dental schools – public, private and total by region.**

| a. Regions | Dental Schools | | | |
| --- | --- | --- | --- | --- |
|  | Private | Public | Total | Response |
| SEA | 307 | 74 | 381 | 8/11 |
| EUR | 81 | 121 | 202 | 27/53 |
| EMR | 62 | 60 | 122 | 6/21 |
| WRP | 58 | 44 | 102 | 16/27 |
| AMR | 33 | 29 | 62 | 10/35 |
| AFR | 13 | 48 | 61 | 33/47 |
| Total | 554 | 376 | 930 | 100/194 |

**Table S5b. Distribution of dental schools – public, private and total by income status.**

| b. Income Status | Dental Schools | | | |
| --- | --- | --- | --- | --- |
|  | Private | Public | Total | Response |
| High | 94 | 160 | 254 | 36/61 |
| Upper-Middle | 77 | 73 | 150 | 19/53 |
| Lower-Middle | 366 | 129 | 495 | 32/49 |
| Low | 17 | 14 | 31 | 13/29 |
| Total | 554 | 376 | 930 | 100/192 |
