# Supplementary material for: Health workforce for oral health inequity: Opportunity for action
Source: PLoS One. 2024 Jun 13;19(6):e0292549. doi: 10.1371/journal.pone.0292549 (PMC11175420; doi:10.1371/journal.pone.0292549)
Supplement: Attachment — Submitted filename: Response to reviewers 20th April and 30th June 2023 .pdf [file pone.0292549.s008.pdf]

Faculty of
Dentistry, Oral &
Craniofacial
Sciences

Centre for Host-Microbiome Interactions

King's College
London Dental
Institute
2nd Floor Dental

30th June 2023

The Editor

PLOS ONE Journal

Dear Sir/Madam,

Manuscript PONE-D-22-35221: **Oral Health Workforce Inequity: opportunity for action**

Our thanks to you as Editor, and your thoughtful reviewers, for your comments and suggestions on the above paper. We have considered them all and have outlined our response to each point in the text below. We feel that the additional work suggested, together with some further refining of the text, has enhanced this paper, and trust that you will agree. Please do not hesitate to contact us if you require anything further.

We have uploaded two versions of the paper one clean and one with highlighted changes as requested. We have made a summary dataset available in line with your requirements as a supplementary file.

We look forward to hearing from you in due course when you have had time to consider this revised manuscript.

Kind regards,

Professor Jennifer E Gallagher MBE
Newland-Pedley Professor of Oral Health Strategy/Honorary Consultant in Dental Public Health

On behalf of co-authors

A. Editorial comments

- A marked-up copy of your manuscript that highlights changes made to the original version has been uploaded as a separate file labeled 'Revised Manuscript with Track Changes'.
- An unmarked version of your revised paper without tracked changes is labeled 'Manuscript'.
    - Both versions uploaded, as requested.

If you would like to make changes to your financial disclosure, please include your updated statement in your cover letter. Guidelines for resubmitting your figure files are available below the reviewer comments at the end of this letter.

- N/A.

When submitting your revision, we need you to address these additional requirements.

1. Please ensure that your manuscript meets PLOS ONE's style requirements, including those for file naming. The PLOS ONE style templates can be found at

https://journals.plos.org/plosone/s/file?id=wjVg/PLOSOne_formatting_sample_main_body.pdf and

https://journals.plos.org/plosone/s/file?id=ba62/PLOSOne_formatting_sample_title_authors_affiliations.pdf

- We trust that we have followed PLOS ONW style requirements in the revised version of the manuscript.

2. We note that you have indicated that data from this study are available upon request. PLOS only allows data to be available upon request if there are legal or ethical restrictions on sharing data publicly. For more information on unacceptable data access restrictions, please see http://journals.plos.org/plosone/s/data-availability#loc-unacceptable-data-access-restrictions.

- Dataset provided as a supplementary file

In your revised cover letter, please address the following prompts:

a) If there are ethical or legal restrictions on sharing a de-identified data set, please explain them in detail (e.g., data contain potentially sensitive information, data are owned by a third-party organization, etc.) and who has imposed them (e.g., an ethics committee). Please also provide contact information for a data access committee, ethics committee, or other institutional body to which data requests may be sent.

b) If there are no restrictions, please upload the minimal anonymized data set necessary to replicate your study findings as either Supporting Information files or to a stable, public repository and provide us with the relevant URLs, DOIs, or accession numbers. For a list of acceptable repositories, please see http://journals.plos.org/plosone/s/data-availability#loc-recommended-repositories.

- Option B has been taken and data uploaded as S4

We will update your Data Availability statement on your behalf to reflect the information you provide.

- Thank you.

3. We note that you have stated that you will provide repository information for your data at acceptance. Should your manuscript be accepted for publication, we will hold it until you provide the relevant accession numbers or DOIs necessary to access your data. If you wish to make changes to your Data Availability statement, please describe these changes in your cover letter and we will update your Data Availability statement to reflect the information you provide.

- Not required as a data set is now available as a supplementary file S4.

4. Please include a separate caption for each figure in your manuscript.

- Captions for figures have been inserted in the manuscript, as requested.

5. Please include captions for your Supporting Information files at the end of your manuscript, and update any in-text citations to match accordingly. Please see our Supporting Information guidelines for more information: http://journals.plos.org/plosone/s/supporting-information.

- Captions for supplementary files have been inserted at the end of the manuscript, as requested.

6. Please upload a copy of Supporting Information Figures 1Sa,b and Tables 1Sa,b which you refer to in your text on page 14 and 11.

- Uploaded as requested.

B. Reviewer #1: Yes

Comments to the Author

1. Is the manuscript technically sound, and do the data support the conclusions?

The manuscript must describe a technically sound piece of scientific research with data that supports the conclusions. Experiments must have been conducted rigorously, with appropriate controls, replication, and sample sizes. The conclusions must be drawn appropriately based on the data presented.

Reviewer #1: Yes

---

2. Has the statistical analysis been performed appropriately and rigorously?

Reviewer #1: Yes

---

3. Have the authors made all data underlying the findings in their manuscript fully available?

The PLOS Data policy requires authors to make all data underlying the findings described in their manuscript fully available without restriction, with rare exception (please refer to the Data Availability Statement in the manuscript PDF file). The data should be provided as part of the manuscript or its supporting information, or deposited to a public repository. For example, in addition to summary statistics, the data points behind means, medians and variance measures should be available. If there are restrictions on publicly sharing data—e.g. participant privacy or use of data from a third party—those must be specified.

- Data set available as a supplemental file S4.

---

4. Is the manuscript presented in an intelligible fashion and written in standard English?

PLOS ONE does not copyedit accepted manuscripts, so the language in submitted articles must be clear, correct, and unambiguous. Any typographical or grammatical errors should be corrected at revision, so please note any specific errors here.

Reviewer #1: Yes

---

5. Review Comments to the Author

Please use the space provided to explain your answers to the questions above. You may also include additional comments for the author, including concerns about dual publication, research ethics, or publication ethics. (Please upload your review as an attachment if it exceeds 20,000 characters)

Reviewer #1: PONE-D-22-35221
Oral Health Workforce Inequity: opportunity for action
PLOS ONE

Dear Authors,
Your manuscript is really interesting and well conducted, and I only have 2 comments in the Methods (see below). The rest of the paper looks fine to me.

- Thank you.

METHODS
Page 7, lines 178-181: I suggest that you add a description in this paragraph of what type of descriptive analysis you performed to report on OHWF density. Measures of frequency, central tendency, variation, position?

- we used the data provided by OHWF accounts (Dentists (per 10 000) (who.int)) where available and replicated this where data were used from the global survey. A sentence has been added to the text to identify that the numerator is the sum of country number of dentists or wider OHWF, divided by the denominator that is the population number and reported per 10,000 population.

Page 7, lines 181-182: Please provide more details on the thematic analysis approach that you took. There are multiple unique guides in the literature for performing a thematic analysis, which one(s) did you uses? Please cite this source. Reading your results, I could not tell if you performed a thematic analysis or a qualitative content analysis. Please review the steps you took to perform the qualitative analysis, and add 1-2 sentences detailing your methods for this analysis.

- Thematic analysis of the open responses was undertaken, and brief description has been added to the Methods.

1. Submit Figures as a high-resolution PPTX or TIF file.
    - Figures are in TIF.

2. Study design and Period is not clear and it is good to say more on it.
    - Further details added to the methods section.

3. ‘‘Density - Global OHWF per 10,000 population, by category and region, 2019’’ Add a picture so that the reader can get a clearer picture of the distribution.
    - We can refer to the global statistics in the baseline report and global maps.

4. Attach the questionnaire used in the study, and cite its reference, and elaborate in detail on its validity and reliability.
    - The questionnaire was already attached and available in the appendix; however, this is now clearer because we have listed the supplementary files (labelled) at the end of the paper
    - This questionnaire was developed in collaboration with WHO teams (oral health and human resources for oral health) against standard criteria outlined in the national workforce accounts, together with the wider literature and was tested with dental and WHO workforce experts re face validity. This is now outlined in the text.
    - Limitations are discussed.

5. Figure 3 elaborates the challenges related to the global oral health work force over the regions. Describe these in a table with statistical analysis to highlight factor/s which significantly challenges among the lists.
    - Thank you for this suggestion. We have looked at the data by region and income status and considered that the analysis by income status provided important insights in relation to each of the challenges and potential solutions and show some important trends. We have therefore replaced Figures 3 and 4 with tables which we consider enhance the content of this paper.
