# Supplementary material for: Health workforce for oral health inequity: Opportunity for action
Source: PLoS One. 2024 Jun 13;19(6):e0292549. doi: 10.1371/journal.pone.0292549 (PMC11175420; doi:10.1371/journal.pone.0292549)
Supplement: Attachment — Submitted filename: PLOS.docx [file pone.0292549.s007.docx]

1. Submit Figures as a high-resolution PPTX or TIF file.
2. Study design and Period is not clear and it is good to say more on it.
3. ‘‘Density - Global OHWF per 10,000 population, by category and region, 2019’’ Add a picture so that the reader can get a clearer picture of the distribution.
4. Attach the questionnaire used in the study, and cite its reference, and elaborate in detail on its validity and reliability.
5. Figure 3 elaborates the challenges related to the global oral health work force over the regions. Describe these in a table with statistical analysis to highlight factor/s which significantly challenges among the lists.
